# Development and Optimization of Ciprofloxacin HCl-Loaded Chitosan Nanoparticles Using Box–Behnken Experimental Design

**DOI:** 10.3390/molecules27144468

**Published:** 2022-07-13

**Authors:** Noha M. Soliman, Faiyaz Shakeel, Nazrul Haq, Fars K. Alanazi, Sultan Alshehri, Mohsen Bayomi, Ahmed S. M. Alenazi, Ibrahim A. Alsarra

**Affiliations:** Department of Pharmaceutics, College of Pharmacy, King Saud University, Riyadh 11451, Saudi Arabia; noha.soliman206@gmail.com (N.M.S.); nhaq@ksu.edu.sa (N.H.); afars@ksu.edu.sa (F.K.A.); salshehri1@ksu.edu.sa (S.A.); bayomimohsen@gmail.com (M.B.); aalenazi1@ksu.edu.sa (A.S.M.A.)

**Keywords:** ciprofloxacin HCl, Box–Behnken design, chitosan nanoparticles, desirability function, response surface methodology, antibacterial effects

## Abstract

Various chitosan (CS)-based nanoparticles (CS-NPs) of ciprofloxacin hydrochloride (CHCl) have been investigated for therapeutic delivery and to enhance antimicrobial efficacy. However, the Box–Behnken design (BBD)-supported statistical optimization of NPs of CHCl has not been performed in the literature. As a result, the goal of this study was to look into the key interactions and quadratic impacts of formulation variables on the performance of CHCl-CS-NPs in a systematic way. To optimize CHCl-loaded CS-NPs generated by the ionic gelation process, the response surface methodology (RSM) was used. The BBD was used with three factors on three levels and three replicas at the central point. Tripolyphosphate, CS concentrations, and ultrasonication energy were chosen as independent variables after preliminary screening. Particle size (PS), polydispersity index (PDI), zeta potential (ZP), encapsulation efficiency (EE), and in vitro release were the dependent factors (responses). Prepared NPs were found in the PS range of 198–304 nm with a ZP of 27–42 mV. EE and drug release were in the range of 23–45% and 36–61%, respectively. All of the responses were optimized at the same time using a desirability function based on Design Expert^®^ modeling and a desirability factor of 95%. The minimum inhibitory concentration (MIC) of the improved formula against two bacterial strains, *Pseudomonas aeruginosa* and *Staphylococcus aureus*, was determined. The MIC of the optimized NPs was found to be decreased 4-fold compared with pure CHCl. The predicted and observed values for the optimized formulation were nearly identical. The BBD aided in a better understanding of the intrinsic relationship between formulation variables and responses, as well as the optimization of CHCl-loaded CS-NPs in a time- and labor-efficient manner.

## 1. Introduction

Pharmaceutical nanoparticles (NPs) are ultrafine colloidal particles with a size ranging from 10 to 1000 nm that contain drugs and exhibit distinct properties from their source materials [1]. Antimicrobial-loaded NPs, for example, can enter cells via endocytosis and release the medicine, thereby eliminating microbe-induced intracellular infections [2]. NPs have a number of advantages, including the capacity to extend drug release. For example, in ocular delivery, they have been observed to enhance the time spent on the corneal surface, boosting ocular bioavailability and lowering systemic toxicity. They also allow lower drug concentrations to be employed in the formulation while generating high drug concentrations at the site of action by enhancing bioavailability [3]. Because of their small size, NPs are particularly well suited to this task, as particles smaller than 10 nm often cause no or very little discomfort [4,5]. Another element to examine before the commercial production of particle systems is stability [6].

Ciprofloxacin hydrochloride (CHCl) is a broad-spectrum antibiotic that belongs to the second generation of fluoroquinolones and has a low rate of spontaneous bacterial resistance. It is slightly soluble in water, while insoluble in popular solvents such as dimethyl sulphoxide, dichloromethane, and acetone [7]. Oral CHCl has a 69 percent absolute bioavailability, a protein binding of less than 30%, and a large distribution volume, which indicates the drug’s ability to be broadly dispersed in tissues [8]. It works against Gram-positive (*Staphylococcus aureus*) as well as Gram-negative (*Escherichia coli*) germs. CHCl works by blocking the enzymes DNA gyrase (topoisomerase II) and topoisomerase IV, which are required for separating bacterial DNA and preventing cell division [9]. It is used as a single agent to treat *S. aureus*-caused topical ocular infections such as conjunctivitis and keratitis. It has been approved for use as a topical therapy for corneal ulcers [10].

As previously reported, many parameters such as CS concentration or tripolyphosphate (TPP) content, pH of the CS solution, and temperature during the cross-linking process are thought to have a substantial impact on the formulation of CHCl-loaded CS-NPs [11]. Despite the ease of the ionic gelation preparation method, due to the huge number of known factors that influence the formulation process, managing the process for reproducible systematic production and the prediction of particle size and surface charge is a difficult issue. The effects of changes in CS molar mass, CS to TPP mass ratio, and solution pH value on NP size, surface charge intensity, and particle aggregation propensity were studied in detail [12]. Optimal conditions for the formulation of NPs can be attained by examining the influence of the variables (at various levels) and their interactions.

Several nanoparticulate drug delivery systems of CHCl such as solid lipid nanoparticles (SLNs) [13,14,15], cationic SLNs [16], PLGA NPs [17,18], calcium carbonate NPs [1,19], CS-NPs [15,20,21,22], hydroxyapatite NPs [23], nanofibers [24] and protein NPs [15] have been studied for therapeutic delivery and to enhance antimicrobial efficacy in the literature. Nevertheless, the Box–Behnken design (BBD)-supported statistical optimization of NPs of CHCl has not been performed in the reported literature. Hence, the aim of this study was to develop CHCl-loaded CS-NPs in order to obtain statistically optimized formulation and to enhance its antibacterial activity compared with pure CHCl. BBD was applied to statistically optimize the formulation in order to minimize the particle size, minimize the PDI for uniform monodispersed particles with a zeta potential of 30–40 mV for a stable dispersion, maximize the entrapment efficiency and obtain a sustained release profile of the drug.

## 2. Materials and Methods

### 2.1. Materials

CHCl was obtained as a kind gift from Medical Union Pharmaceuticals (Ismailia, Egypt). High-purity, low-molecular weight CS from crab shells in the form of powder, with a degree of deacetylation of 75–85%, and TPP were purchased from Sigma Aldrich (San Diego, CA, USA). Potassium chloride, sodium chloride, sodium phosphate monobasic and potassium phosphate monobasic were obtained from Fisher Scientific (Fair Lawn, NJ, USA), as the chemicals required to obtain phosphate buffer (PB) media for release studies. Methanol and acetonitrile (HPLC grades) and acetic acid (glacial) were acquired from BDH Laboratory Supplies (Poole, UK). Muller–Hinton broth was supplied by Oxoid (Basingstoke, UK). The bacterial strains *P. aeruginosa* ATCC^®^ 27853 and *S. aureus* ATCC^®^ 25923 were obtained from Pharmaceutical Microbiological Laboratory (King Saud University, Riyadh, Saudi Arabia). Purified water was used throughout, and it was obtained by means of a Milli-Q^®^ water purifier (Millipore, Molsheim, France). All chemicals were of pharmaceutical and analytical grade and were used without further purification.

### 2.2. Experimental Design

For optimizing CS-NPs and evaluating the association between responses and variables, the response surface methodology (RSM) was applied. The goal was to maximize a response influenced by a number of different independent variables. As the number of factors expanded, BBDs were developed to limit the sample size. These contained a subset of the factorial combinations from the 3^*k*^ factorial design [25] and provided three levels for each component. The number of experiments (N) needed to produce BBD was calculated using the following formula:

(1) N=2k (k−1)+Co

where *k* represents the number of factors and *C_o_* is the number of central points. Only three levels (−1, 0, +1) of adjustment were required for all factor levels, with evenly spaced intervals between them. Table 1 shows the coded factor levels for a BBD of a three variable system. In this study, the BBD was chosen to investigate the relationship between the response functions (PS, PDI, ZP, EE and drug release) and the independent variables (TPP concentration, CS concentration and ultra-sonication energy) on three different levels (Table 2).

### 2.3. Preparation of CS-TPP NPs by Ionic Gelation

The CS-NPs were made using a modified ionic gelation technique as described in the literature [26]. The CS solution (0.3–1 mg/mL) was stirred overnight at room temperature using a magnet stirrer after being dissolved in 0.25 percent (*v*/*v*) acetic acid. TPP was dissolved in water at a concentration of 0.1–0.6 mg/mL. Both the CS and TPP solutions were blended at room temperature with continual magnetic stirring at 500 rpm. To eliminate insoluble particles, the CS solution was filtered through a Millipore filter (pore size 0.45 µm). To make CS-NPs, the TPP was added dropwise to a CS solution at 900 rpm for 10 min. When TPP solution was added to the CS solution, a drug-free nanodispersion of CS-TPP NPs developed spontaneously. This was performed at room temperature with continual magnetic stirring. CHCl was dissolved in 2 mL of water at a drug to carrier weight ratio of 0.5:1 to make CHCl-loaded NPs. The drug solution was then dropped into the CS solution during magnetic stirring, followed by the addition of the TPP solution drop by drop at room temperature at 900 rpm for 90 min, after which the solution was treated for further particle size reduction using an ultrasonication probe (tip diameter: 13 mm) as previously reported [27]. To extract the generated NPs, the solution was centrifuged for 60 min at 13,500× *g*. After that, the prepared NPs were freeze-dried.

### 2.4. Characterization of CHCl-Loaded CS-NPs

After centrifugation, the supernatant was discarded and the NPs were re-suspended in purified water for size and morphological analysis.

#### 2.4.1. Determination of PS, PDI and ZP

Dynamic light scattering (DLS) on the Zetasizer Nano ZS (Malvern Instruments, Worcestershire, UK) was used to measure the mean PS (Z-average), PDI, and ZP of CS-TPP NPs in the hydrated state. To produce an average, light scattering was measured in triplicate at a 90° angle and at a temperature of 25 °C. On the Zetasizer Nano ZS, the ZP was calculated using an electrophoretic light scattering approach. The NP samples were diluted with 0.001 M KCl before being placed in the electrophoretic cell. The mean electrophoretic mobility data were used to determine the ZP values [28].

#### 2.4.2. Determination of Morphology

The size and shape of CS-NPs were studied using a transmission electron microscope (TEM). A drop of the CS-NP dispersion was applied to carbon-coated copper grids, allowed to dry at room temperature, and then analyzed without any further modification or negative staining [26].

#### 2.4.3. Differential Scanning Calorimetry (DSC) Study

Thermal analysis was utilized to evaluate any physical or chemical interactions between the medication and the excipients. DSC thermograms for roughly 5 mg of samples (pure medicine and excipients) were acquired using aluminum pans with lids in a dynamic nitrogen environment (20 mL/min), at a heating rate of 10 °C/min, and a temperature range of 50–400 °C. Indium (melting point 156 °C) was used to calibrate the DSC cell.

### 2.5. Lyophilization

The supernatant was decanted after the NPs were generated and centrifuged, and the separated NPs were resuspended in a 5% mannitol solution before being freeze dried for 24 h at a maximum temperature of −40 °C and a maximum vacuum of 100 × 10^−3^ mbar to obtain dry CHCl-loaded CS-NPs.

### 2.6. High Performance Liquid Chromatography (HPLC) Assay of CHCl

CHCl was determined using the HPLC technique. CHCl was separated using an internal standard of acebutolol and a mobile phase of methanol-acetonitrile-acetic acid (5%) in a volume ratio of 6:7:87 at a flow rate of 1.2 mL/min at an ambient temperature using a Symmetry C stainless steel column (150 mm length × 3.9 mm i.d, 5 µm particle size). Fluorescence detection at 280 nm (excitation) and 455 nm (emission) at an attenuation of 2 and a gain of 10 was used to monitor the column effluent. Volumes of 20 µL were injected into the chromatographic system from aliquots loaded in the autosampler tray [29].

### 2.7. Determination of EE

The difference between the entire amount added to the loading solution and the amount of non-entrapped CHCl left in the supernatant was used to estimate the amount of CHCl entrapped and adsorbed. The NPs were disseminated in 25 mL of phosphate buffer pH 7.4 (PB) and centrifuged at 13,500× *g* for 30 min at 10 °C. The samples were filtered through a 0.2 µm membrane filter, and the amount of CHCl was measured using the abovementioned HPLC procedure. The EE was calculated using the following formula:

(2) $$EE\;\left(\%\right)=\left(\frac{W1-W2}{W1}\right)\times 100$$

where *W*1 is the weight of total of drug and *W*2 is the amount of free drug measured in the supernatant.

### 2.8. In Vitro Release Study of CHCl

The in vitro release of CHCl from NPs was studied using a static Franz diffusion cell. The cylindrical donor compartment’s terminal portion was fitted with a cellulose acetate membrane (MWCO: 12–14 kDa). The donor compartment received an aliquot of 1 mL of the NPs distributed in PB. The receptor compartment was filled with 12 mL of PB solution, which was kept at 37 °C using a magnetic stirrer. At predetermined intervals, 1 mL aliquots were extracted and replaced with the same volume of fresh PB. The release of CHCl from CS-NPs was studied for 8 h. The amount of drug released was assessed using the HPLC method at λ excitation at 280 nm and λ emission at 455 nm [29].

### 2.9. Antibacterial Study

A broth microdilution M7-A8 method approved by the Clinical Laboratory Standards Institute-CLSI (previously NCCLS) was used to establish the minimum inhibitory concentration (MIC). In Muller–Hinton (MH) broth, the CHCl, blank CS-NPs, and CHCl-loaded NPs were gradually diluted. The CHCl, blank CS-NPs, and CHCl-loaded NPs were gradually diluted in Muller–Hinton (MH) broth. Due to its insolubility, CS was diluted in MH broth, which included 0.25 percent (*v*/*v*) acetic acid. To produce a bacterial concentration of 1 × 10^5^ CFU/mL, bacterial strains were injected. After 24 h of incubation at 37 °C, the MIC was determined to be identical to the concentration in the tube without observable growth. For each bacterial species, the test was repeated three times [30]. The MIC of the strain was determined by a value that was agreed upon on two or more occasions. CHCl was used as a positive control, while the blank control wells merely had MH broth and bacterial inoculum [31].

## 3. Results

### 3.1. CHCl NPs Formulation

The ionic gelation process of CS with TPP was used to create CHCl-loaded CS-NPs. The TPP is a multivalent polyanion with low toxicity and cost, and unlike other crosslinkers, it has few handling and storage restrictions [32]. The formation of CS-NPs is based on crosslinking between positively charged amino groups of CS and negatively charged polyanions of TPP at both the inter- and intramolecular levels. The CS solution spontaneously changed from a clear to opalescent solution with the slow addition of polyanion solutions [32]. Increasing the CS concentration resulted in a significant increase in PS. Furthermore, raising the polyanion weight ratio resulted in a rise in the NPs’ process yield. The turbidity of the dispersions increased as a result of this effect. Further increases in the polyanion ratio, on the other hand, led in particle aggregation, most likely due to a shift in the ZP of the NPs towards lower values. Because the PS is a major predictor of the drug-loaded NPs’ biological activity, the experimental conditions were changed to allow for the synthesis of NPs with a size close to 200 nm. Because CS is a weak base polyelectrolyte insoluble at neutral and alkaline pH [33], the lyophilized CS-NPs generated in the experiment have a white powdery form and are insoluble in water, dilute acidic, and alkaline solutions. The degree of protonation of CS is largely determined by the pH of the solution. According to Shu and Zhu (2002), the protonation degree of CS declined fast from 100 percent to 0 percent when the pH of the CS solution climbed from 4.7 to 8, showing that there is a critical pH at which CS begins to deprotonate [34].

### 3.2. Experimental Design Summary

Using RSM and the three-level, three-factorial BBD developed with Design Expert^®^ software (version 7), the process factors impacting PS, PDI, EE, cumulative release after 8 h, and ZP were investigated. TPP concentrations of 0.1–0.6 mg/mL, CS concentrations of 0.3–1 mg/mL, and ultrasonication energy of 10–40 Watt were the variable input parameters, with factor levels coded as −1 (low), 0 (middle), and 1 (high), respectively. For statistical calculations, the three independent variables were labeled A (TPP concentration), B (CS concentration), and C (ultra-sonication energy). Table 3 shows the range and levels employed in the experiments. To optimize the process parameters, 15 runs were undertaken, and experiments were carried out according to the real experimental design matrix. Table 4 presents a summary of the measured responses.

### 3.3. Characterization of CHCl-Loaded CS-NPs

Zetasizer was used to examine the mean PS and size distribution of each batch of NP suspension. A typical batch of NPs with a mean diameter of 250 nm and a narrow size distribution (PDI < 0.5) is represented by the size distribution profile. Large PDI values are most likely due to NP aggregation. The explanation for the aggregation is because CS is a self-adhesive hydrogel polymer that, due to strong inter-particle contacts, prefers to create bigger particles [15]. As a result of their Brownian motion in aqueous solution, these NPs interact with one another to form clusters. As a result, the average diameter of interacting particles is always bigger than the actual diameter. Surface charge, or ZP, has a significant impact on particle stability in a suspension due to electrostatic repulsion between particles. It can also determine how NPs interact with cell membranes in vivo, which are typically negatively charged.

**Figure 1:**
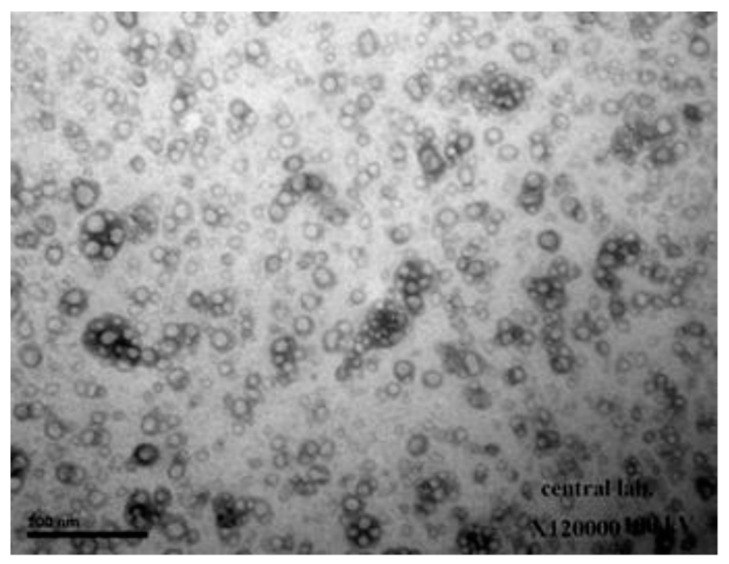
TEM image of ciprofloxacin hydrocholride (CHCl)-loaded chitosan (CS) nanoparticles (NPs).

When seen with TEM, NPs seemed to be significantly smaller than the average particle size observed with DLS (Figure 1). The smallest population has an average diameter of at least 198 nm, according to DLS scaling.

**Figure 2:**
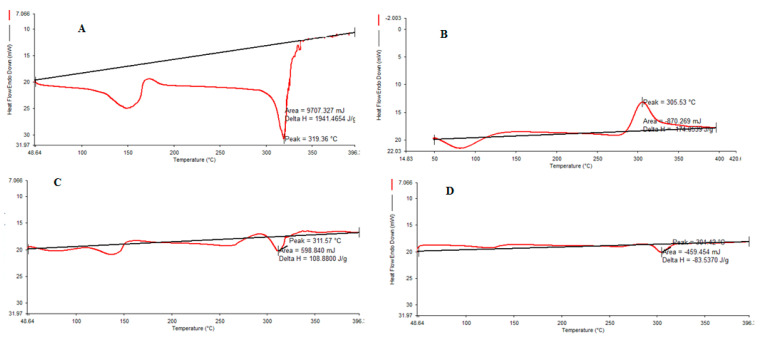
Differential scanning calorimetry (DSC) thermogram of (**A**) pure CHCl, (**B**) CS, (**C**) CHCl-CS physical mixture and (**D**) NPs.

DSC thermograms of pure CHCl, pure CS, CHCl-CS physical mixture and CHCl-loaded NPs are presented in Figure 2A–D. The DSC thermograms show that pure CHCl presented two endothermic peaks (Figure 2A). The first peak is at 150 °C, which shows water evaporation, and the sharp peak at 319 °C corresponds to the melting point (Figure 2A), where the decomposition process was followed by the melting of the drug. This sharp peak was dramatically reduced in the DSC profiles of cross-linked CHCl-loaded CS-NPs (Figure 2D). CS, on the other hand, demonstrated an endothermic peak at temperatures below 100 °C and an exothermic peak at temperatures above 305 °C (Figure 2B). Exothermic peaks are caused by the degradation of polyelectrolytes due to dehydration and depolymerization events, most likely due to partial decarboxylation of the protonated carboxylic groups and oxidation reactions of the polyelectrolytes. In a CHCl-CS physical mixture at a 1:1 weight ratio, the second endothermic peak shifted to the lower temperature of 304.42 °C (Figure 2C). Cross linking produced by decreased crystallinity resulted in a change in the solid-state structure of CS.

### 3.4. Fitting of Data to the Selected Model

All of the observed responses were fitted to first-order, second-order, and quadratic models at the same time, and the model was validated using the ANOVA, lack of fit, and R^2^ tests. The statistical significance of the ratio of mean square variation due to regression and mean square residual error was tested using ANOVA. The large value of F suggests that the regression equation can explain the majority of the variation in the response. A positive value in the regression equation for a response suggests a factor–response relationship that encourages optimization (synergistic impact), whereas a negative value shows an inverse link (antagonistic effect). The model’s failure to describe data in the experimental domain at points not included in the regression is measured by the lack of fit. One of the desirable statistical parameters to establish model fitting on the answers is insignificant lack of fit. The amount of variation in a response variable that can be explained by its link with one or more predictor variables is known as R^2^. R^2^ is a measure of how well a model fits the data. The higher the R^2^, the better the model fits the data. The R^2^ value is always between 0 and 100%. By fitting the data for observed responses to several models, it was discovered that the best fitted model for three of the dependent variables, namely PS, EE, and release, was a quadratic model, while ZP fit into a linear model and PDI fit into a two-factor interaction model. The impacts of these parameters and their relative relevance on the EE of nanoparticulate systems are represented by the coefficients for CS, TPP, and ultra-sonication energy. The quadratic (non-linear) character of the relationship is indicated by higher values of the standard error (SE) for coefficients.

**Figure 3:**
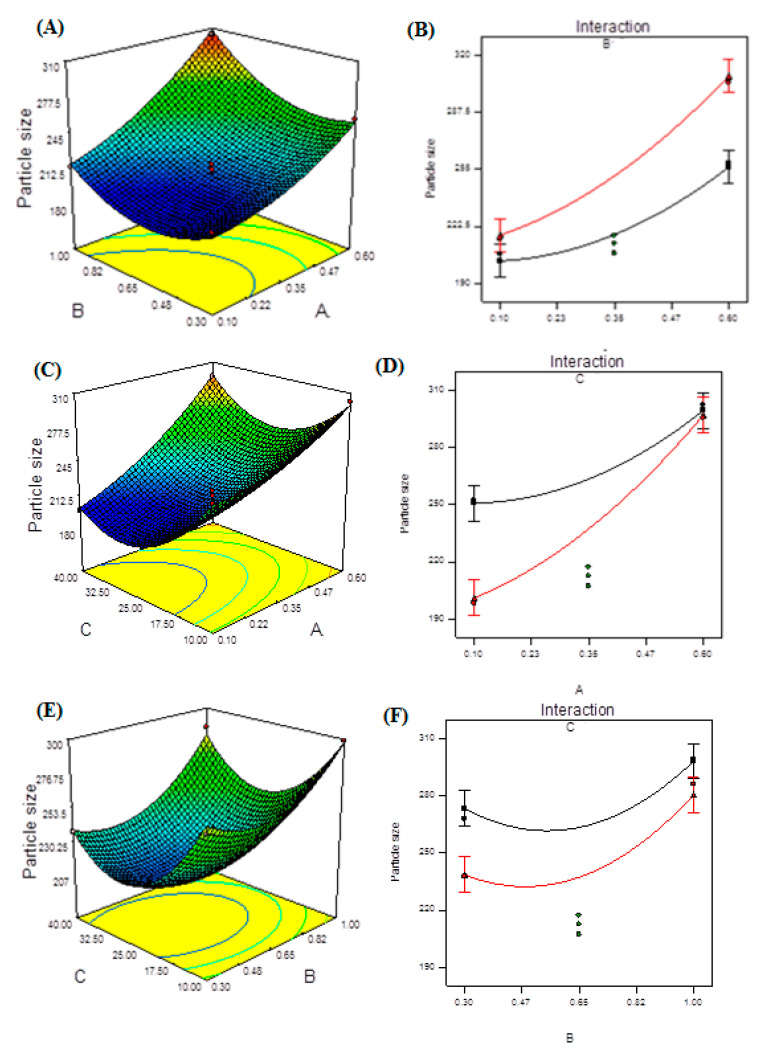
Model graphs: particle size. (**A**) Three-dimensional response surface plots showing the effect of variables (A: TPP concentration and B: CS concentration) on response (particle size), (**B**) interaction plot, (**C**) 3D response surface plots showing the effect of variables (A: TPP concentration and C: ultrasonication energy) on response (particle size), (**D**) interaction plot, (**E**) 3D response surface plots showing the effect of variables (B: CS concentration and C: ultrasonication energy) on response (particle size) and (**F**) interaction plot.

The measured particle size of NPs at each level for each tested independent variable is shown in Supplementary Figure S1. All measured responses of NPs are summarized in Table 5. Figure 3 demonstrates the 3D plots and their interaction plots of the BBD model. Table S1 illustrates that the response fits to a quadratic regression model equation. The ANOVA summary for quadratic model is presented in Table 6. The model F-value of 69.51 indicates that the model is significant, according to Table 6. Due to noise, there is only a 0.01 percent chance that this large model F-value will occur. Model terms are important when prob > F is less than 0.0500. A, B, C, AB, AC, A^2^, B^2^, and C^2^ are important model terms in this situation. The lack of fit F-value of 1.61 indicates that the lack of fit has no bearing on the pure error. There is a 40.46 percent risk that noise will cause a lack of fit F-value. For the model to fit, a minor lack of fit is desired. Table S2 showed that the predicted R^2^ of 0.9050 is in reasonable agreement with the adjusted R^2^ of 0.9778. The ratio of 22.190 indicates an adequate signal. Figure S3 shows the measured ZP at each level for each tested independent variable. Table S3 illustrates that the response fits to a linear regression model equation. Table 7 shows that the model’s F-value of 16.56 indicates that it is significant. Due to noise, there is only a 0.02 percent chance that this large model F-value will occur. The model term A was important in this scenario. The lack of fit F-value of 5.78 indicates that the lack of fit is insignificant in comparison to the pure error. There is a 15.63 percent probability that noise will cause a lack of fit F-value. For the model to fit, a minor lack of fit is desired.

The predicted R^2^ of 0.6196 in Table S4 is reasonably close to the adjusted R^2^ of 0.7692. The signal-to-noise ratio of 11.798 suggests that the signal is adequate. The assumption of normality of error terms is tested in Figure S4A. Our presumption of normalcy is correct. Figure S4B is a representation of actual response values versus anticipated response values that aids in detecting a value or group of values that the model is unable to predict. Figure S4C is a useful tool for determining the power law transformation to use. No transformation was recommended for this model. Figure S4D helps to detect outliers in the data points. No outlier points were found for this model. The influence of varied values of the independent variables TPP, CS, and sonication on the measured response ZP is shown in Figure S5. Figure S6 shows the measured PDI at each level for each tested independent variable. Table S5 illustrates that the response fits to a two-factor interaction regression model equation.

The model F-value of 14.11 indicates that the model is significant, as seen in Table 8. Due to noise, there is only a 0.07 percent chance that this large model F-value will occur. A, C, AC, and BC are important model terms in this situation. The lack of fit F-value of 7.18 indicates that the lack of fit has no bearing on the pure error. There is a 12.73 percent risk that noise will cause a lack of fit F-value. For the model to fit, a minor lack of fit is desired. The predicted R^2^ of 0.5885 is not as near to the adjusted R^2^ of 0.8489 as one might expect, as seen in Table S6. This could happen as a result of outliers or other factors. The ratio of 12.403 suggests that the signal is adequate. The data diagnosis graph in Figure S7A verifies the assumption of normality of the error terms. Our presumption of normalcy is correct. Figure S7B is a representation of actual response values versus anticipated response values that aids in detecting a value or group of values that the model is unable to predict. Figure S7C is a useful tool for determining the power law transformation to use. Figure S7D helps to detect outliers in the data points.

**Figure 4:**
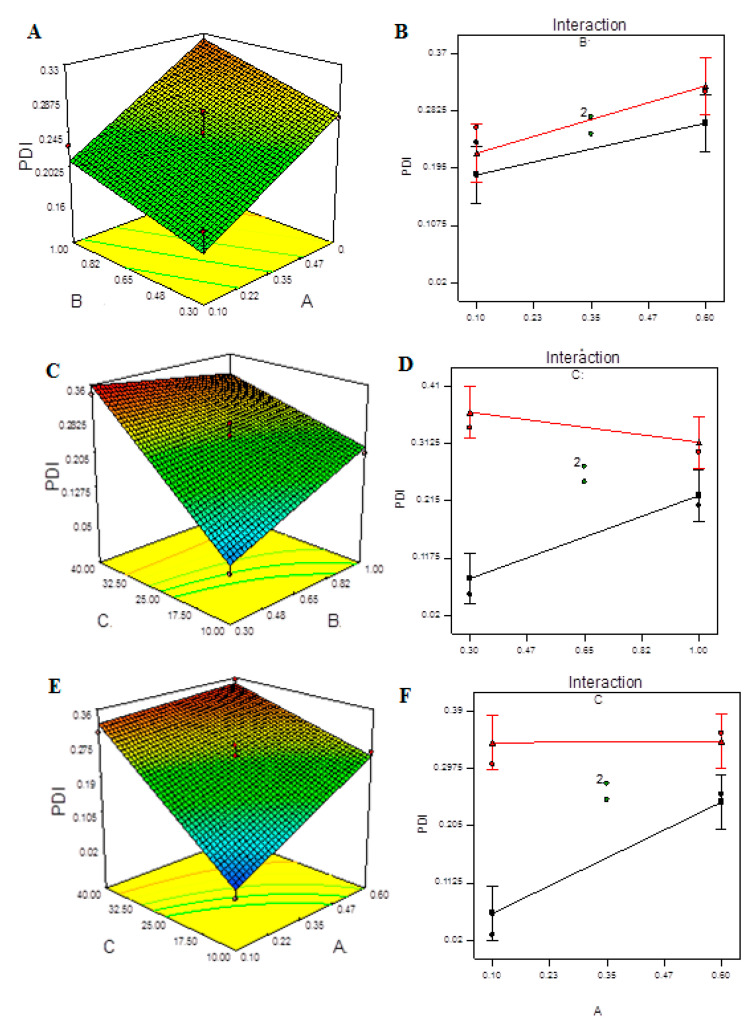
Model graphs: PDI. (**A**) Three-dimensional response surfaces plots showing the effect of variables (A: TPP concentration and B: CS concentration) on response on response (PDI), (**B**) interaction plot, (**C**) 3D response surfaces plots showing the effect of variables (B: CS concentration and C: ultrasonication energy) on response (PDI), (**D**) interaction plot, (**E**) 3D response surfaces plots showing the effect of variables (A: TPP concentration and C: ultrasonication energy) on response (PDI) and (**F**) interaction plot.

Figure 4 demonstrates the 3D plots and their interaction plots in the BBD model. It is obvious from the plots that increasing the ultra-sonication input above a certain energy value caused an increase in the PDI due to re-agglomeration of the smaller particles formed upon treatment. The nonlinear structure of the 3D response surfaces and their related contour plots reveals that the independent factors had significant interactions and mutually dependent influences on the response. Interaction plots depict how the response varies when two variables are altered at the same time while the third variable remains constant at its central level. Figure S8 depicts the measured EE for each tested independent at each level. The quadratic model provides the best fit for the data and can explain the effect of the independent variables on the response, as shown in Table S7. Table 9 shows that the model’s F-value of 822.61 indicates that it is significant. Due to noise, there is only a 0.01 percent chance that this large model F-value will occur. A, C, AB, AC, A^2^, B^2^, and C^2^ are important model terms in this situation.

**Figure 5:**
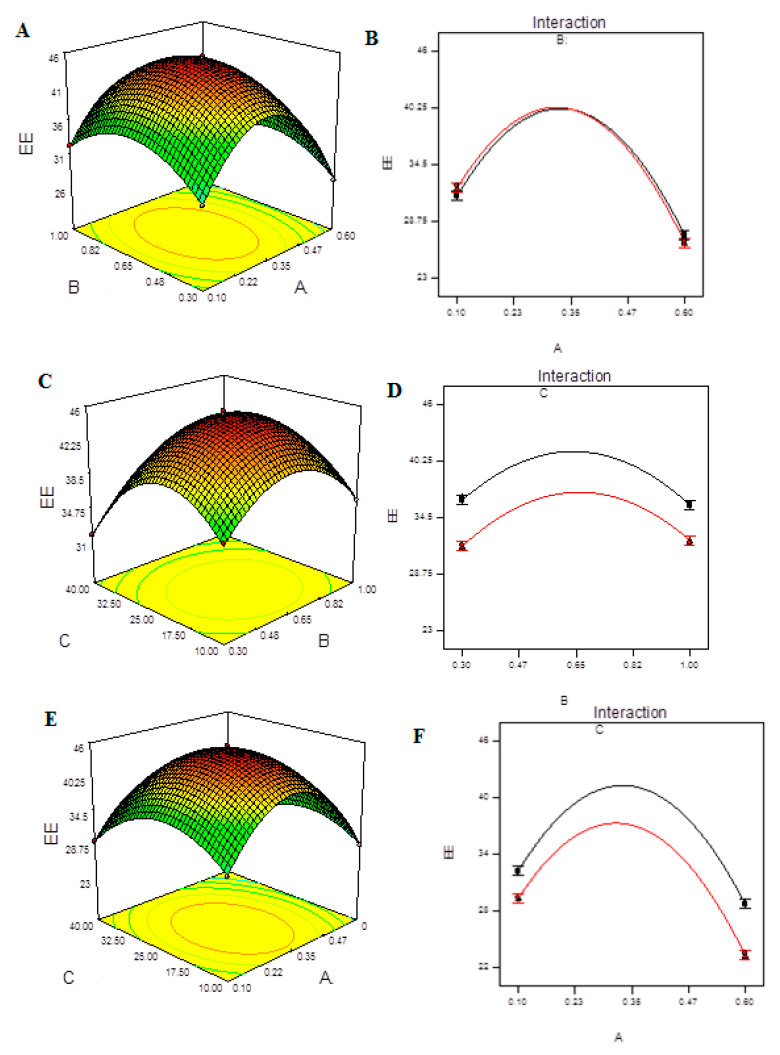
Model graphs: EE. (**A**) Three-dimensional response surfaces plots showing the effect of variables (A: TPP concentration and B: CS concentration) on response on response (EE), (**B**) interaction plot, (**C**) 3D response surfaces plots showing the effect of variables (B: CS concentration and C: ultrasonication energy) on response (EE). (**D**) interaction plot, (**E**) 3D response surfaces plots showing the effect of variables (A: TPP concentration and C: ultrasonication energy) on response (EE) and (**F**) interaction plot.

The lack of fit F-value of 2.56 indicates that the lack of fit has no bearing on the pure error. A lack of fit F-value owing to noise has a 29.37 percent chance of occurring. For the model to fit, a minor lack of fit is desired. The predicted R^2^ of 0.9911 is in reasonable agreement with the adjusted R^2^ of 0.9981, as shown in Table S8. The signal-to-noise ratio of 89.304 indicates a good signal. The assumption of normality of error terms is tested in Figure S9A. In this, our assumption of normality is valid. The predicted vs. actual plot (Figure S9B) quantitatively compares the findings of the experimental response values with the predicted values from the developed models. The Box–Cox transformation (Figure S9C) seeks a suitable exponent (lambda) to improve the normalcy of positively or negatively skewed variables and determines whether or not the response should be assessed on a different scale. Figure S9D aids in the detection of data point outliers. Response surface diagrams for the EE of CHCl-loaded CS-NPs are shown in Figure 5A,C,E. According to the results, the EE was higher with intermediate concentrations of either CS or TPP and mild ultra-sonication treatment. The interaction effect of A, B and C on EE can be seen in the interaction plots in Figure 5B,D,F. Only the interactions AB and AC had a significant effect on the EE of CS-NPs.

**Figure 6:**
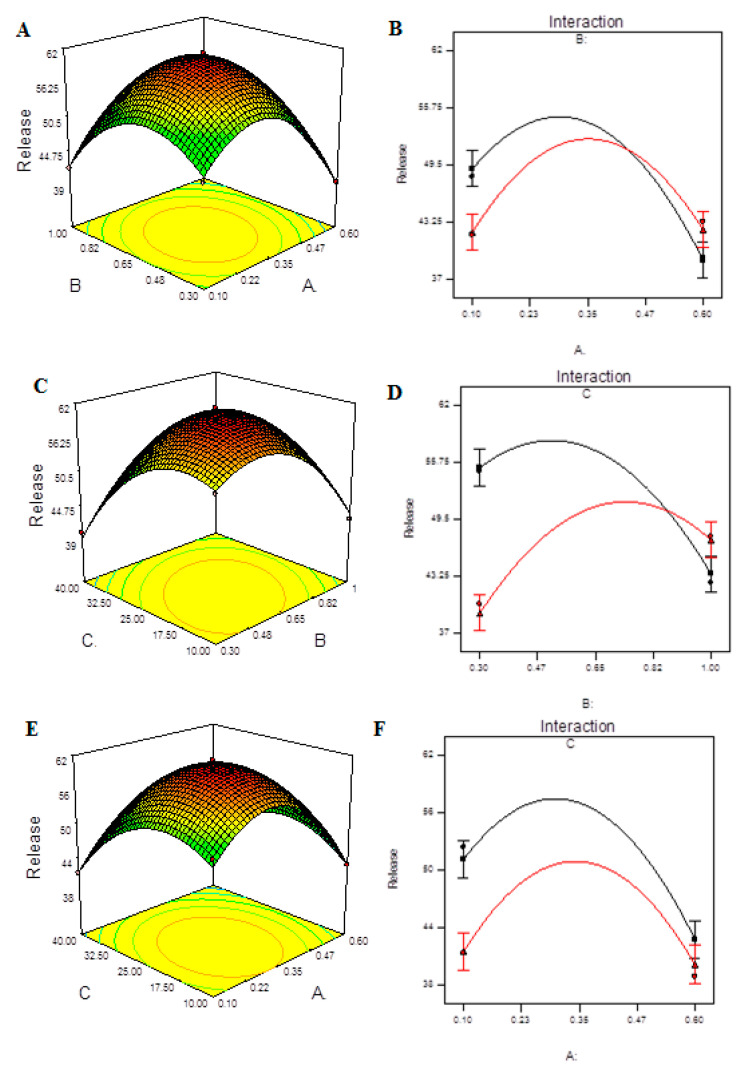
Model graphs: release of CHCl. (**A**) Three-dimensional response surfaces plots showing the effect of variables (A: TPP concentration and B: CS concentration) on response on response (release), (**B**) interaction plot, (**C**) 3D response surfaces plots showing the effect of variables (B: CS concentration and C: ultrasonication energy) on response on response (release), (**D**) interaction plot, (**E**) 3D response surfaces plots showing the effect of variables (A: TPP concentration and C: ultrasonication energy) on response (release) and (**F**) interaction plot.

Figure S10 shows the measured release of CHCl at each level for each tested independent variable. The quadratic model provides the best fit for the data and can explain the effect of the independent variables on the response, as shown in Table S9. The model F-value of 66.91 indicates that the model is significant, as seen in Table 10. Due to noise, there is only a 0.01 percent chance that this large model F-value will occur. In this case, A, C, AB, AC, BC, A^2^, B^2^, C^2^ are significant model terms. The lack of fit F-value of 8.15 indicates that the lack of fit has no bearing on the pure error. A lack of fit F-value owing to noise has an 11.12 percent chance of occurring. For the model to fit, a minor lack of fit is desired. The predicted R^2^ of 0.8768 is in reasonable agreement with the adjusted R^2^ of 0.9769, as reported in Table S10. The signal-to-noise ratio of 21.424 indicates a good signal. The diagnosis plots are shown in Figure S11. Figure S11A verifies the assumption of error term normality. Our assumption of normalcy is correct in this circumstance. Figure S11B is a representation of actual response values versus anticipated response values that aids in detecting a value or group of values that the model is unable to predict. Figure S11C is a useful tool for determining the power law transformation to use. Figure S11D aids in the detection of data point outliers. The 3D response surface plots are shown in Figure 6A,C,F. It was observed that the release of CHCl from NPs was higher with intermediate concentrations of either CS or TPP and mild ultra-sonication treatment. The interaction effect of A, B and C on release can be seen in Figure 6B,D,F). All of the interactions showed a significant effect on the release of CHCl from the NPs.

### 3.5. Optimized Formula of CHCl NP

**Figure 7:**
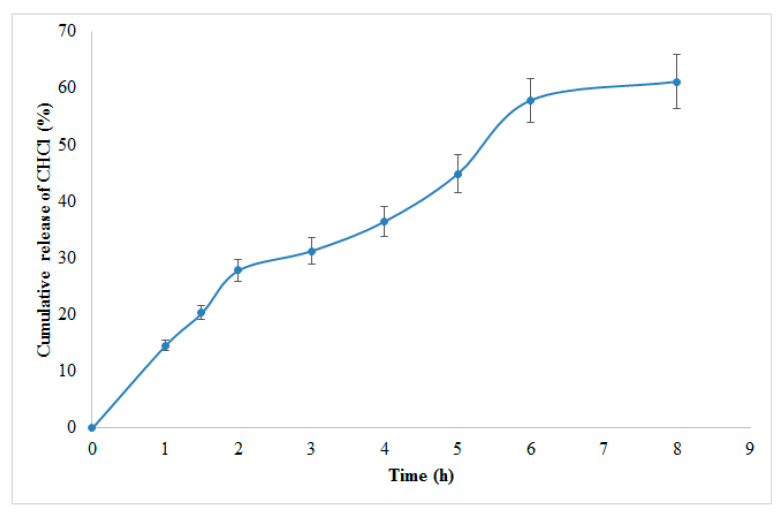
CHCl percent amount released from the optimized NPs in phosphate buffer (pH 7.4, 37 °C).

Based on Design Expert^®^ (version 7) modeling and a desirability factor of 95%, the program recommended the following elements for the preparation of the ideal formulation: 0.25 mg/mL TPP concentration, 0.65 mg/mL CS, and a 25-watt sonication input (Table S11). Figure 7 shows the release profile of CHCl from optimized NPs. The improved formulation was then created, and all of the required evaluations for PS, PDI, ZP, EE, and 8-h release were completed. To calculate the % prediction error, the experimental values of the answers were quantitatively compared to the projected values (Table S12).

### 3.6. In Vitro Drug Release

**Figure 8:**
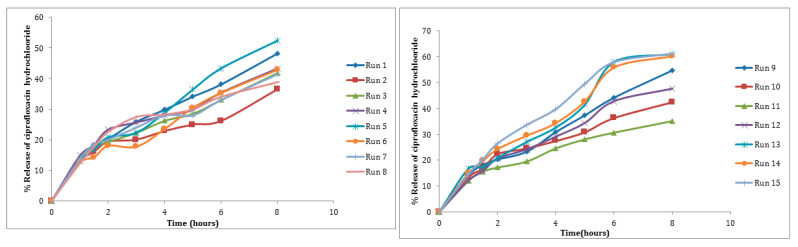
Percent amount released of CHCl from NPs in phosphate buffer (pH 7.4, 37 °C).

For the release of CHCl among the independent variables, selected B was found to be insignificant (*p* > 0.05). Several attempts have been made to improve drug encapsulation and release time, with the first burst release of the drug being the main issue. This is partly due to the CS/TPP NPs’ poor mechanical strength, and the first burst release can be minimized by increasing the particle’s mechanical strength [34]. In vitro release experiments revealed a profile with two nearly distinct stages when plotting the percent drug released vs. time (Figure 8). Figure 6 demonstrates the 3D plots and their interaction plots in the BBD model for in vitro drug release. The in vitro release profile of CHCl from an optimized formulation is presented in Figure 7.

### 3.7. Antibacterial Study

**Figure 9:**
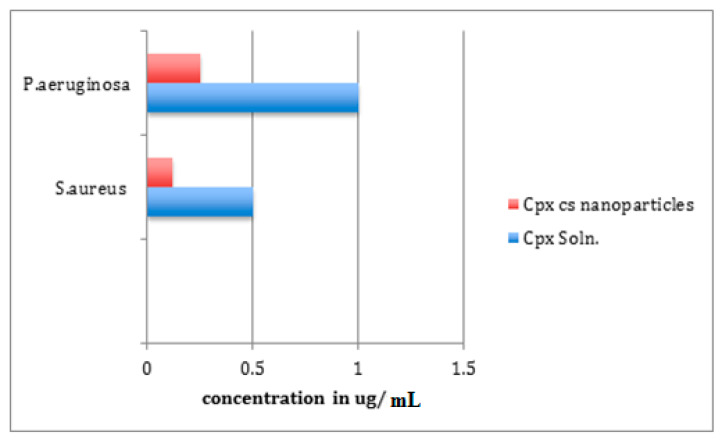
Antibacterial activity of CHCl soln. and CHCl-loaded CS-NPs.

Figure 9 shows the results of the antibacterial activity of the improved formulation. For *S. aureus* and *P. aeruginosa*, the determined MIC for the CHCl solution was 0.5 µg/mL and 1 µg/mL, respectively. The optimized NPs’ MICs were 0.12 µg/mL in the case of *S. aureus* and 0.25 µg/mL in the case of *P. aeruginosa*. This large difference (above four times decrease in MIC) indicated the superior properties of the NP formulation, as shown in Figure 9 and Table 11.

## 4. Discussion

The amino groups of CS are protonated at a low pH (3.5), resulting in a decreased charge density of the molecules. This results in insufficient TPP cross-linking and, as a result, bigger particle sizes. The deprotonation degree of TPP increases as pH rises, but the protonation degree of CS is unaffected. As a result, at pH 5.5, the PS drops considerably. At pH 6.0, the particle size increased dramatically, indicating that the degree of protonization at the particle surface was reduced, resulting in particle aggregation. ZP showed the same declining tendency at pH 5.5 and a rise at pH 6 [35]. The pH was kept at 5.5 for all runs based on these findings and similar studies in the literature. Under the right conditions, particles with a smaller size distribution can be created. Even if the NPs are generated under optimal conditions, they become unstable with time and the particles clump together. For future usage, NPs are normally freeze-dried or preserved in buffer solutions. The NP suspension is chilled in freeze-drying devices, resulting in the creation of pure water ice crystals. The remaining solution becomes increasingly concentrated in terms of NPs as water crystallizes, and its viscosity rises as it cools. Despite the fact that freeze-drying removes water from the NP suspension, it may result in several stresses such as freezing, mechanical, and dehydration stress. The stability of the NPs may be affected by these stressors [36]. Cryoprotectants and lyoprotectants are commonly employed to counteract the freezing process’ destabilizing effects and to maintain NPs’ functional qualities and activities after freeze-drying [37]. Cryoprotectants can fix NPs in a glassy matrix, inhibiting the formation of NP agglomerates and the mechanical stress of ice crystals during freeze-drying [38]. They also have preservation effects against variations in ionic strength of the NP suspension. The type and amount of cryoprotectant utilized, on the other hand, should be considered. Agglomeration may occur if there is an overabundance of protectant. The addition of trehalose, mannitol, sorbitol, and glycerol to silica NPs during freeze-drying, according to Sameti et al. [39], is an effective technique to maintain the physical properties of silica NPs. A series of studies were performed to apply the BBD, with the goal of investigating alternative combinations of parameters and analyzing the combined effects of these elements. The BBD model does not contain simultaneous combinations of all factors at their maximum or lowest values, and thus can avoid extreme treatment combinations in particular [40]. The coefficient of determination (R^2^), analysis of variance (ANOVA), and response plots were used to analyze the results. The most extensively used second-order polynomial equation devised to fit experimental data and find relevant model terms is RSM, which can be expressed as

(3) $$Y=\beta_{0}+\sum^{\;}\beta_{i}x_{i}+\sum^{\;}\beta_{ii}x_{ii}^{2}+\sum^{\;}\beta_{ij}x_{i}x_{j}+\varepsilon$$

where *Y* is the predicted response, *β*_0_ is the constant coefficient, *β*_*i*_ is the linear coefficient, *β*_*ii*_ is the quadratic coefficient, *β*_*ij*_ is the interaction coefficients, and *ε* is the error of the model. Because the independent variables in this study were coded as A, B, and C, the equation may be written as [41,42]:

(4) $$Y=\beta_{0}+\beta_{i}A+\beta_{i}B+\beta_{i}C+\beta_{ii}A^{2}+\beta_{ii}B^{2}+\beta_{ii}C^{2}+\beta_{ij}AB+\beta_{ij}AC+\beta_{ij}BC$$

Positive ZP greater than 30 mV is thought to indicate that the colloidal system is stable. The ZP degree can help to determine the colloidal system’s stability. When all of the particles have a large positive or negative ZP (where the positivity and negativity are more than +30 mV and −30 mV, respectively), they repel each other, and the dispersion is stable. When the particles’ ZP values are low, however, there is insufficient force to prevent the particles from aggregating [43]. The dehydration of the CS/TPP hydrogel NPs during sample preparation for TEM imaging can explain the apparent disparity between the two results. DLS also overestimates the size of NPs because it measures the perceived size (hydrodynamic radius) of a particle, which includes hydrodynamic layers that form around hydrophilic particles, such as those made of CS/TPP [44]. The DSC results were similar to those reported by Motwani et al., who found that gatifloxacin-loaded CS-NPs displayed a decrease in intensity and changed to a lower temperature due to the drug’s encapsulation in NPs [44]. However, because the change is not too great, it is safe to believe that the encapsulating process had no effect on the CS polymer’s structure or qualities. The final equation in terms of coded factors is expressed as follows:

(5) $$Particle\;size=+212.40+36.04\times A+16.71\times B-13.05\times C+9.32\times A\times B\;+11.80\times A\times C+4.26\times B\times C+11.67\times A^{2}+22.26\times B^{2}+38.03\times C^{2}$$

where A is the TPP concentration, B is the CS concentration and C is the sonication energy input. The prior equation’s R^2^ value was found to be 0.9921, suggesting an excellent fit. The PS levels measured for the various batches indicated a broad range of results (i.e., values ranged from a minimum of 198 nm to a maximum of 304 nm). The findings show that the variables chosen for the study had a significant impact on the PS score. The large variety of coefficient values for the terms reflects this as well. The average result of altering one variable at a time from a low to a high level is represented by the primary effects of A, B, and C. The interaction terms (AB, AC, BC, A^2^, B^2^, and C^2^) demonstrated how the PS changes when two variables are altered at the same time. The positive coefficients of A and B imply a synergistic effect on PS, whereas the negative value of C implies an antagonistic effect. The protonated amino groups of CS cause electrostatic repulsion between CS molecules in acidic circumstances; meanwhile, there are also inter-chain hydrogen bonding interactions between CS molecules. The intermolecular hydrogen bonding attraction and the intermolecular electrostatic repulsion are at equilibrium below a particular concentration of CS (2.0 mg/mL, as stated) [45]. As CS concentration rises in this range, CS molecules approach each other with a limit, resulting in a restricted increase in intermolecular cross-linking, resulting in larger but still tiny particles. Microparticles develop easily above this concentration, owing to increased hydrogen bonding interactions that result in a large number of CS molecules engaged in the crosslinking of a single particle. Because the electrostatic repulsion between particles is insufficient to preserve the physical stability of these big particles, the production of microparticles frequently results in a flocculent precipitate [11].

With increasing CS concentration, PS increased (from 198 to 304 nm, depending on TPP content, 0.1–0.6 mg/mL). PS varied from 198 nm to 304 nm depending on TPP concentration (0.1 mg/mL to 0.6 mg/mL) at 1 mg/mL CS concentration (Table 5). The PS rose as the concentration of either CS or TPP was raised, according to the findings. Calvo et al. discovered that the production of CS/TPP NPs was limited to certain CS and TPP concentrations [26]. This was also confirmed in our research. It has been observed that the concentration of CS or TPP must be less than 1.5 mg/mL and 1.0 mg/mL, respectively, to avoid the formation of microparticles [11]. The fact that viscosity is increased at increasing concentrations of CS could explain the increase in PS with CS. This causes an increase in viscous forces, which demonstrate resistance to droplet breakdown by stirring and, as a result, a rise in PS [11]. Furthermore, the PS of NPs increased as CS concentration increased, possibly because there were more binding sites available for ionic cross linking of molecules [26]. The effect of TPP concentration on PS was found to be more pronounced in this study than the effect of CS concentration on PS. There would be more TPP ions to cross-link with amino groups on CS chains as TPP concentration increased [26]. Because of distinct degradation methods and varying sizes of degraded CS molecules participating in the ionotropic gelation with TPP molecules, ultrasonic radiation produces different NP sizes. The cavitation effect is the principal mechanism of CS molecule destruction by ultrasonic radiation [46]. According to Lan et al., cavitation processes occur simultaneously throughout the fluid to destroy the polymeric CS molecule or break up the nanoparticle cluster into tiny particles. Sonication had an influence on both the CS molecules and the NP aggregates that formed after the combination was formed [47].

Figure S2A verifies the assumption of normality of the error terms. The majority of the points are concentrated around the red line in this scenario, showing that the error terms are approximately typical. As a result, our presumption of normalcy is correct. A plot of the actual response values versus the expected response is shown in Figure S2B. It aids in the detection of a value or set of values that the model is unable to predict. The data should be evenly distributed across the line. Figure S2C is a useful tool for determining the power law transformation to use. Based on the best lambda value, which is found at the minimum point of the curve created by the natural log of the sum squares of the residuals, a proposed transformation is presented. It was found that there is no recommended transformation for this response. Figure S2D helps to detect outliers in the data points. Outliers are points outside the red line that do not fit the current model well. No outlier points were found for this model.

The utilized program generated three-dimensional (3D) response surface plots (Figure 3A,C,E). The interaction effects of independent variables on responses are depicted in these graphs. The 3D plots show the effects of two factors on a single answer, while the third element remained constant. The 3D curves clearly show how TPP and CS concentrations, as well as ultrasonication, affect PS. Extremely high TPP or CS concentrations resulted in the creation of bigger NPs, which is highly undesirable. Furthermore, as the ultrasonication input was increased, the PS decreased due to the smaller particles generated during treatment. PS was found to have synergistic effects when A, B, and C were combined. The interaction effects of the two components (TPP and CS concentrations) may be seen in Figure 3B by considering ultrasonication as a constant. In terms of coded factors, the final equation is as follows:

(6) Zeta Potential=+34.74−6.14×A+1.62×B−0.063×C

where A is the TPP concentration, B is the CS concentration and C is the sonication energy input.

For ZP, the selected variable (A) showed a significant effect. The negative coefficient of (A) indicates an antagonistic effect on ZP. In terms of surface charge, the samples produced positively charged NPs. When CS and TPP were combined together, they produced compact complexes with an overall positive surface charge, as determined by ZP values in agreement with the literature [48]. At a fixed concentration of CS (0.65 mg/mL), the ZP of NPs fell from 42.2 and 42.3 to 29.9 and 30.5 mV as the TPP concentration increased from 0.1 to 0.6 mg/mL. The TPP concentration had the greatest impact on the ZP of NPs, which ranged from 38.1 to 42.3, 30.2 to 39.6, and 27.3 to 30.5 mV for TPP concentrations of 0.10, 0.35, and 0.6 mg/mL, respectively. With rising TPP concentrations, the availability of protonated CS amine groups decreases. Increased TPP concentration resulted in a decrease in ZP, which is consistent with the literature [49,50]. NPs with a surface charge larger than 30 mV are more stable, according to the literature, and this value is adequate to avoid particle aggregation [40]. It is also worth noting that CS concentration and sonication at different energy levels did not have a significant effect on the ZP, but the relationship between ZP positively correlates with the CS concentration in accordance with the literature [40]. The ZP is a measurement of particle surface charge, which can have a significant impact on particle stability in a suspension due to electrostatic repulsion between particles, similar to the in vivo interaction of NPs [51]. Furthermore, through electrostatic contact with the negatively charged mucin of the mucus, a positive surface charge would provide mucoadhesive properties, enhancing residence time and absorption.

The concentration of TPP reduced as the concentration of ZP grew, which is highly undesirable. While an increase in CS concentration was associated with an increase in ZP, the effect was determined to be minor. It is worth noting that the ZP was unaffected by sonication. When the availability of positively charged –NH2 groups on CS is larger at lower TPP concentrations, the needed minimum value of ZP (more than +30 mV) can be attained for the stability of nanoparticulate suspensions. In terms of coded factors, the final equation is as follows:

(7) $$\begin{array}{ll} PDI & =+0.25+0.045\times A+0.022\times B+0.093\times C+6.000E-003\times A\times B\\ & -0.044\times A\times C-0.048\times B\times C \end{array}$$

where A is the TPP concentration, B is the CS concentration and C is the ultra-sonication energy input. The intended 2FI model shows that the effect of each element on the PDI is not linear, and the ultimate effect is the result of a collection of interactions. Optimization and prediction will be achievable with such a model. It is worth noting that ultra-sonication energy has 2 times the effect on PDI with a positive coefficient of 0.093 as compared to the other significant factors, with coefficients equal to 0.045, −0.044, and −0.048 for A, AC and BC, respectively. The factors A, AC and BC have a similar effect on the PDI. It should be obvious that NP-based colloidal systems have a high tendency to aggregate. The mechanical energy associated with reaction stirring speed could be greater than the electrostatic repulsion energy between NP positive surface charges, resulting in aggregation. This is a common occurrence in the production of solitary NPs [52]. PDI regression analysis suggests that A has a positive effect, while the interactions have a negative effect. According to the literature, dispersions with a PDI of less than 0.3 suggest strong particle size homogeneity [40]. Ultrasonication causes a quick de-aggregation of agglomerates, indicating that smaller particles could be obtained and PS distributions narrowed. It has been hypothesized that ultrasonication can help to regulate PS and PDI. In this regard, it should be emphasized that van der Waals forces exceed repulsive forces in the case of NP agglomeration. As a result of the attractive van der Waals forces, freshly produced NPs clump together. It is also worth noting that ultrasonication has a size-reducing effect at first, but after a critical treatment, it has a negative impact on particle agglomeration [38]. Ultrasonic irradiation causes NPs to de-agglomerate due to ultrasonic cavitation effects. The agglomerates of the NPs can be disrupted by shockwaves, micro-jets, and micro-streaming created by acoustic cavitation dispersing the suspension [38]. When sound waves with undulating high- and low-pressure areas pass through an NP suspension, cavitation bubbles form. High-speed liquid jets and localized hydrodynamic micro-streaming are generated by the inertial collapse of the bubbles, which puts mechanical stress on the attractive forces between individual NPs [40]. The agglomerated particles fragment into smaller particles as a result of these occurrences. Many researchers [27,46,53] reported similar findings. In terms of coded factors, the final equation is as follows:

(8) $$EE=+45.26-2.38\times A+0.016\times B-2.07\times C-0.39\times A\times B-0.64\times A\times C\;+0.27\times B\times C-10.73\times A^{2}-5.17\times B^{2}-6.12\times C^{2}$$

The results indicate a major contributing effect of A, B and C on *EE*. Negative value of the coefficient for A and C (TPP concentration and ultra-sonication energy) indicates a negative effect on *EE*. Particles obtained at high values are lower in *EE* than those obtained at lower values of the variables. Drugs can be loaded into a nanoparticulate system in two ways: during the preparation of the particles or following their formulation. In such systems, the drug molecules are embedded physically into the matrix or adsorbed onto the surface. Drug loading can be increased by integrating the drug into the particle composition. In CS-based particle systems, both water-soluble and insoluble medicines can be loaded. Water-soluble medicines such as CHCl are combined with the CS solution to create a homogenous mixture from which the particles are made [12].

Larger NPs have a higher drug loading capacity in a polydisperse system, whereas smaller NPs are likely to deliver drugs more efficiently to tissues or cells [54]. However, there was an inverse association between PS and CHCl encapsulation, which can be explained by the fact that at larger concentrations of CS and TPP, the polymers make up the majority of the NP matrix, leaving less room for drug encapsulation. The EE of NPs was observed to range from 23.5 to 45.5 percent. The EE of CHCl into CS–TPP NPs was likewise found to be highest (45%) when the CS, TPP, and drug were all employed at intermediate concentrations, but it was lowest (26.72%) when both the CS and TPP were utilized at higher concentration levels. Additionally, according to Osman et al., drug entrapment is caused by interactions between the positive CS amino group and the negative carboxylate group of CHCl, as well as entrapment of the drug in the CS matrix and hydrogen bonding. Thus, increasing the TPP concentration decreases the EE as fewer sites are available to attach to the negative carboxylate group of CHCl [55]. Gajra et al., on the other hand, showed a rise in EE with a corresponding increase in TPP, which could be attributed to improved crosslinking density of the CS matrix as well as more CS molecules participating in the ionic gelation process to create NPs that can accommodate more medication [56]. Stirring has been shown to have a deleterious impact on *EE* in the literature. Because of insufficient encapsulation of the drug in NPs or poor interaction of the drug with CS and TPP, the *EE* decreases as the stirring speed increases, which could explain the unfavorable effect of ultra-sonication on *EE* [57]. In terms of coded factors, the final equation is as follows:

(9) $$Release=+60.92-2.42\times A-0.90\times B-3.06\times C+2.55\times A\times B+1.75\times A\times C\;+4.91\times B\times C-10.05\times A^{2}-7.67\times B^{2}-6.97\times C^{2}$$

The value of R^2^ of the previous equation was found to be 0.9918, indicating good fit.

The quick release behavior is detected in the first phase of the release profile (approximately 15–20 percent of the medication is released within the first hour for all formulations), while the controlled-release nature of the system is confirmed in the second phase [35,40]. Kalam et al. [40] confirmed an initial fast release of CHCl followed by a slower persistent release. Furthermore, the initial burst release of drug was most likely caused by drug release from the surface of CS-NPs. Due to hydration and expansion of the polymer, a steady and extended release of CHCl was discovered subsequently [58].

The acceptable agreement between observed values and software-predicted values, as well as the low error rate, show the validity of all the models, as well as their suitable precision for predicting optimum circumstances in the domain of independent variable levels. CHCl is a carboxylic-acid derivative of quinolone with excellent antibacterial activity against both Gram-positive and Gram-negative bacteria [59]. The positive charge of the amino groups in CS provides antibacterial activity, allowing them to adhere to the bacterial cell surface and interfere with normal membrane processes, preventing bacterial growth [60]. *P. aeruginosa* and *S. aureus* were used to test the antibacterial efficacy. Two of the most frequent ocular infections that cause bacterial infection of the human cornea were chosen [10]. Abdelrahman et al. showed that CHCl-loaded CS-NPs decreased the MIC values of G +ve *B. subtilis* and G–ve *E. coli* in a similar pattern to that found in this research [61], while the control revealed that acetic acid had no extra negative effects on bacterial growth at low concentrations. Because *P. aeruginosa* has a higher MIC than *S. aureus*, greater amounts were required to stop the bacteria from growing. This is consistent with the findings of another investigation [55]. However, in the previously mentioned work, the CHCl-loaded NPs showed similar antibacterial activity to those obtained with a CHCl aqueous solution. This difference in results is probably due to the difference in the bacterial strains used for the antibacterial test, but it also ensures that incorporating CHCl into CS-NPs using TPP as a counter ion does not alter the activity of the drug [55].

CHCl-loaded CS-NPs also had better antibacterial action against Gram-positive bacteria than Gram-negative bacteria according to the findings. This could be due to variations in cell wall structure and content. As previously observed in the literature [62,63], Gram-positive bacteria have a higher negative charge on their cell surface than Gram-negative bacteria, resulting in increased NP adsorption and inhibitory activity. Furthermore, with a MIC of > 4 µg/mL, the data revealed that blank CS-NPs had little antibacterial activity. The CS solution had MIC values of 64 and 128 µg/mL, respectively, against *S. aureus* and *P. aeruginosa*. According to Shi et al. (2006), CS NPs have stronger antibacterial activity than CS powder because the polycationic nano CS has a bigger surface area and charge density and may interact with the negatively charged surface of the bacterial cell to a greater extent [64]. The increased antibacterial activity of tiny NPs may be due to an increase in surface area with decreasing particle size [63]. It is worth noting that CHCl-loaded CaCO_3_ NPs had the same antibacterial activity as untreated CHCl against the *S. aureus* test organism, as described in the literature [19]. Similarly, Dillen et al. (2006) found no significant changes in the MIC values of CHCl-loaded Eudragit/PLGA NPs and untreated CHCl in their study [65]. This suggests that using CS polymer to encapsulate antibacterial drugs improves the antibacterial activity of the total formula better than other inert polymers.

## 5. Conclusions

Several nanoparticulate drug delivery systems of CHCl have been investigated for therapeutic delivery and to enhance antimicrobial efficacy. However, the BBD-supported statistical optimization of CS-NPs of CHCl has not been performed in the literature. Hence, the aim of this study was to develop CHCl-loaded CS-NPs in order to obtain a statistically optimized formulation and to enhance its antibacterial activity compared with pure CHCl. Many factors are included during the preparation of CS-NPs. Changing one variable at a time to assess the influence of the variables on responses is a time-consuming, costly, and challenging approach. The application of RSM, as detailed in this work, yields valuable results for the major impacts and interactions among the elements studied, as well as graphical representations of intricate relationships. The effect of the independent variables—linear, quadratic, and interactions—on the chosen responses within the constraints of minimizing the PS, and maximizing the encapsulation of a drug and sustaining its release as predicted using polynomial equations, was explained using a three-factor, three-level BBD for the optimization of the prepared NPs. The BBD made it easier to understand the connection between formulation variables and answers. It was demonstrated that by studying the effects of the parameters, the NPs could be tailored to meet the requirements of a certain drug delivery system. Furthermore, the ideal design could be predicted, and the results matched the theoretical values. Preparation of CS-NPs using the ionic gelation method yielded well-sized positively charged CS-NPs with acceptable PDI, good *EE* and drug release. The produced CS-NPs have a steady dispersive character, as demonstrated by high ZP values. The optimized NPs can act as scaffolds for post studies using these NPs as a model system for encapsulation of hydrophilic or hydrophobic drugs. The optimized formula showed superior antibacterial activity against two bacterial strains, which confirms its potential suitability for ocular application to treat bacterial infections. The prospect of enhanced antimicrobial activity of CHCl with CS was realized not only through crosslinking and the added antibacterial properties of CS, but also through nanosized particles that were adjusted.

## Figures and Tables

**Table 1:** Coded factor levels for a Box–Behnken design (BBD) of a three-variable system.

Experiment	*x*_1_	*x*_2_	*x*_3_
1	−1	−1	0
2	1	−1	0
3	−1	1	0
4	1	1	0
5	−1	0	−1
6	1	0	−1
7	−1	0	1
8	1	0	1
9	0	−1	−1
10	0	1	−1
11	0	−1	1
12	0	1	1
C	0	0	0
C	0	0	0
C	0	0	0

**Table 2:** List of independent variables and responses used in the design.

Independent Variables	Levels			Response	Constrains
	−1	0	+1		
TPP concentration (mg/mL)	0.1	0.35	0.6	Particle size	Minimize
				PDI	<0.5
CS concentration (mg/mL)	0.3	0.65	1	Zeta potential	30–40 mV
				Encapsulation efficiency	Maximize
Ultra-sonication energy (watt)	10	25	40	Release type	Sustained Release

**Table 3:** Experimental design summary of independent variables.

Factor	Name	Units	Type	Low Actual	High Actual	Low Coded	High Coded	Mean
A	TPP concentration	mg/mL	Numeric	0.100	0.60	−1.000	1.000	0.350
B	Chitosan concentration	mg/mL	Numeric	0.30	1.00	−1.000	1.000	0.650
C	Sonication input	watt	Numeric	10.00	40.00	−1.000	1.000	25.000

**Table 4:** Experimental design summary of responses.

Response Name	Obs	Analysis	Minimum	Maximum	Mean	STDEV	Transformation	Model
Particle size	15	Polynomial	198.200	304.500	250.777	±38.356	None	Quadratic
PDI	15	Polynomial	0.029	0.354	0.242	±0.090	None	2FI
ZP	15	Polynomial	27.300	42.3	35.160	±5.827	None	Linear
EE	15	Polynomial	23.5	45.500	33.609	±7.750	None	Quadratic
Release	15	Polynomial	38.900	61.300	47.751	±7.925	None	Quadratic

**Table 5:** Box–Behnken experimental design showing independent variables with measured responses (* center points).

Std. Order	A	B	C	PS	PDI	ZP	EE	% Cumulative CHCl Release at 24 h
1	0.10	0.30	25	206.8 ± 12.3	0.234 ± 0.019	41.8 ± 1.3	31.23 ± 2.87	48.2 ± 5.73
2	0.60	0.30	25	258.7 ± 13.2	0.265 ± 0.04	27.3 ± 0.5	27.12 ± 4.08	39.43 ± 8.63
3	0.10	1.00	25	215.3 ± 8.6	0.257 ± 0.011	38.1 ± 1.6	32.38 ± 2.56	41.86 ± 5.75
4	0.60	1.00	25	304.5 ± 22.2	0.312 ± 0.016	27.6 ± 0.75	26.72 ± 5.78	43.3 ± 2.6
5	0.10	0.65	10	252.4 ± 9.7	0.029 ± 0.13	42.2 ± 3.2	32.05 ± 1.30	52.4 ± 4.58
6	0.60	0.65	10	302.4 ± 12.8	0.256 ± 0.091	29.9 ± 0.9	28.7 ± 5.74	42.9 ± 2.54
7	0.10	0.65	40	198.2 ± 16.9	0.304 ± 0.026	42.3 ± 1.6	29.4 ± 5.89	41.4 ± 2.50
8	0.60	0.65	40	295.4 ± 9.7	0.354 ± 0.05	30.5 ± 2.3	23.5 ± 4.87	38.9 ± 7.31
9	0.35	0.30	10	267.9 ± 25.3	0.056 ± 0.131	39.6 ± 1.1	36.57 ± 2.75	54.75 ± 5.83
10	0.35	1.00	10	299.06 ± 11.3	0.207 ± 0.021	30.6 ± 0.7	35.72 ± 1.67	42.55 ± 3.71
11	0.35	0.30	40	237.8 ± 16.9	0.339 ± 0.091	39.8 ± 2.1	31.68 ± 5.40	40.18 ± 2.47
12	0.35	1.00	40	286 ± 7.7	0.298 ± 0.061	30.8 ± 1.2	31.91 ± 1.56	47.63 ± 4.62
* 13	0.35	0.65	25	217.3 ± 2.3	0.273 ± 0.087	33.2 ± 2.3	45.2 ± 2.56	61.16 ± 6.42
* 14	0.35	0.65	25	207.2 ± 1.7	0.257 ± 0.068	34.3 ± 1.1	45.5 ± 1.74	60.3 ± 7.12
* 15	0.35	0.65	25	212.7 ± 212.7 ± 2.6	0.247 ± 0.09	35.5 ± 1.7	45.08 ± 1.36	61.3 ± 6.40

**Table 6:** ANOVA for the response surface quadratic model.

Source	Sum of Squares	df	Mean Square	F Value	*p*-Value Prob > F	
Model	21,892.71	9	2432.52	69.51	0.0001	Significant
A	10,389.61	1	10,389.61	296.89	<0.0001	
B	2233.12	1	2233.12	63.81	0.0005	
C	1361.38	1	1361.38	38.90	0.0016	
AB	347.82	1	347.82	9.94	0.0253	
AC	556.96	1	556.96	15.92	0.0104	
BC	72.59	1	72.59	2.07	0.2093	
A2	502.64	1	502.64	14.36	0.0128	
B2	1829.16	1	1829.16	52.27	0.0008	
C2	5340.82	1	5340.82	152.62	<0.0001	
Residual	174.97	5	34.99			
Lack of Fit	123.83	3	41.28	1.61	0.4046	not significant
Pure Error	51.14	2	25.57			
Cor Total	22,067.68	14				

**Table 7:** ANOVA for the response surface linear model.

Source	Sum of Squares	df	Mean Square	F Value	*p*-Value Prob > F	
Model	322.51	3	107.50	16.56	0.0002	significant
A	301.35	1	301.35	46.41	<0.0001	
B	21.12	1	21.12	3.25	0.0987	
C	0.031	1	0.031	4.813 × 10^−3^	0.9459	
Residual	71.43	11	6.49			
Lack of Fit	68.78	9	7.64	5.78	0.1563	not significant
Pure Error	2.65	2	1.32			
Cor Total	393.94	14				

**Table 8:** ANOVA for response surface two factor interaction model.

Source	Sum of Squares	df	Mean Square	F Value	*p*-Value Prob > F	
Model	0.11	6	0.018	14.11	0.0007	significant
A	0.016	1	0.016	12.98	0.0070	
B	4.050 × 10^−3^	1	4.050 × 10^−3^	3.19	0.1119	
C	0.070	1	0.070	54.95	<0.0001	
AB	1.440 × 10^−4^	1	1.440 × 10^−4^	0.11	0.7449	
AC	7.832 × 10^−3^	1	7.832 × 10^−3^	6.17	0.0379	
BC	9.216 × 10^−3^	1	9.216 × 10^−3^	7.26	0.0273	
Residual	0.010	8	1.269 × 10^−3^			
Lack of Fit	9.704 × 10^−3^	6	1.617 × 10^−3^	7.18	0.1273	not significant
Pure Error	4.507 × 10^−4^	2	2.253 × 10^−4^			
Cor Total	0.12	14				

**Table 9:** ANOVA for response surface quadratic model.

Source	Sum of Squares	df	Mean Square	F Value	*p*-Value Prob > F	
Model	669.76	9	74.42	822.61	<0.0001	significant
A	45.22	1	45.22	499.86	<0.0001	
B	2.113 × 10^−3^	1	2.113 × 10^−3^	0.023	0.8845	
C	34.24	1	34.24	378.46	<0.0001	
AB	0.60	1	0.60	6.64	0.0496	
AC	1.63	1	1.63	17.97	0.0082	
BC	0.29	1	0.29	3.22	0.1325	
A2	424.91	1	424.91	4696.93	<0.0001	
B2	98.69	1	98.69	1090.93	<0.0001	
C2	138.29	1	138.29	1528.69	<0.0001	
Residual	0.45	5	0.090			
Lack of Fit	0.36	3	0.12	2.56	0.2937	not significant
Pure Error	0.094	2	0.047			
Cor Total	670.21	14				

**Table 10:** ANOVA for response surface quadratic model.

Source	Sum of Squares	df	Mean Square	F Value	*p*-Value	
Model	934.24	9	103.80	66.91	0.0001	significant
A	46.71	1	46.71	30.10	0.0027	
B	6.52	1	6.52	4.20	0.0957	
C	74.97	1	74.97	48.32	0.0009	
AB	26.06	1	26.06	16.80	0.0094	
AC	12.25	1	12.25	7.90	0.0376	
BC	96.53	1	96.53	62.22	0.0005	
A^2^	372.93	1	372.93	240.37	<0.0001	
B^2^	217.36	1	217.36	140.10	<0.0001	
C^2^	179.38	1	179.38	115.62	0.0001	
Residual	7.76	5	1.55			
Lack of Fit	7.17	3	2.39	8.15	0.1112	not significant
Pure Error	0.59	2	0.29			
Cor Total	942.00	14				

**Table 11:** MIC values for two bacterial strains.

	*S. aureus* ATCC 25923	*P. aeruginosa* ATCC 27853
CHCL soln.	0.5 ug/mL	1 ug/mL
CHCl CS-NPs	0.12 ug/mL	0.25 ug/mL

## Data Availability

This study did not report any data.

## Supplementary Materials

The following supporting information can be downloaded at: https://www.mdpi.com/article/10.3390/molecules27144468/s1, Figure S1: Particle size distribution at different levels of the independent variables (A) TPP (mg/mL) (B) CS (mg/mL) and (C) ultrasonication energy (Watt).; Figure S2: Diagnosis plots: particle size. (A) Normal plot of residuals and (B) regression line of response surface plot of actual versus predicted value; (C) Box–Cox plot for power transforms and (D) externally studentized residuals vs. run number; Figure S3: Zeta potential distribution at different levels of the independent variables: (A) TPP (mg/mL), (B) CS (mg/mL) and (C) ultrasonication energy (Watt); Figure S4: Diagnosis plots; zeta potential. (A) Normal plot of residuals and (B) regression line of response surface plot of actual versus predicted value; (C) Box–Cox plot for power transforms and (D) externally studentized residuals vs. run number; Figure S5: Effect of variables on zeta potential (A: top left), TPP 24 concentration (B: bottom right), CS concentration and (C: bottom left) ultrasonication energy; Figure S6: PDI distribution at different levels of the independent variables: (A) TPP (mg/mL), (B) CS (mg/mL) and (C) ultrasonication energy (Watt); Figure S7: Diagnosis plots: PDI. (A) Normal plot of residuals and (B) regression line of response surface plot of actual versus predicted value; (C) Box–Cox plot for power transforms and (D) externally studentized residuals vs. run number; Figure S8: EE distribution at different levels of the independent variables: (A) TPP (mg/mL), (B) CS (mg/mL) and (C) ultrasonication energy (Watt); Figure S9: Diagnosis plots: EE. (A) Normal plot of residuals and (B) regression line of response surface plot of actual versus predicted value; (C) Box–Cox plot for power transforms and (D) externally studentized residuals vs. run number; Figure S10: Release distribution at different levels of the independent variables: (A) TPP (mg/mL) (B) CS (mg/mL) and (C) ultrasonication energy (Watt); Figure S11: Diagnosis plots: release of CHCl. (A) Normal plot of residuals and (B) regression line of response surface plot of actual versus predicted value; (C) Box–Cox plot for power transforms and (D) externally studentized residuals vs. run number; Table S1: Model summary statistics; response: particle size; Table S2: Summary of the results of the regression analysis after fitting to the quadratic model; Table S3: Model summary statistics; response: zeta potential; Table S4: Summary of the results of the regression analysis after fitting to the linear model; Table S5: Model summary statistics; response: PDI; Table S6: Summary of the results of the regression analysis after fitting to the two factor interaction model; Table S7: Model summary statistics; response encapsulation efficiency; Table S8: Summary of the results of the regression analysis after fitting to the quadratic model; Table S9: Model summary statistics; response: release; Table S10: Summary of the results of the regression analysis after fitting to the quadratic model; Table S11: Optimized formula variables; Table S12: Predicted vs. actual responses obtained for the optimized formulation.

## Sample Availability

Samples of the compounds CHCl are available from the authors.
