# Supplementary material for: Development and Optimization of Ciprofloxacin HCl-Loaded Chitosan Nanoparticles Using Box–Behnken Experimental Design
Source: Molecules. 2022 Jul 13;27(14):4468. doi: 10.3390/molecules27144468 (PMC9321140; doi:10.3390/molecules27144468)
Supplement: Supplementary file 1 [file molecules-27-04468-s001.zip › molecules-1773253-supplementary.pdf]

Supplementary materials

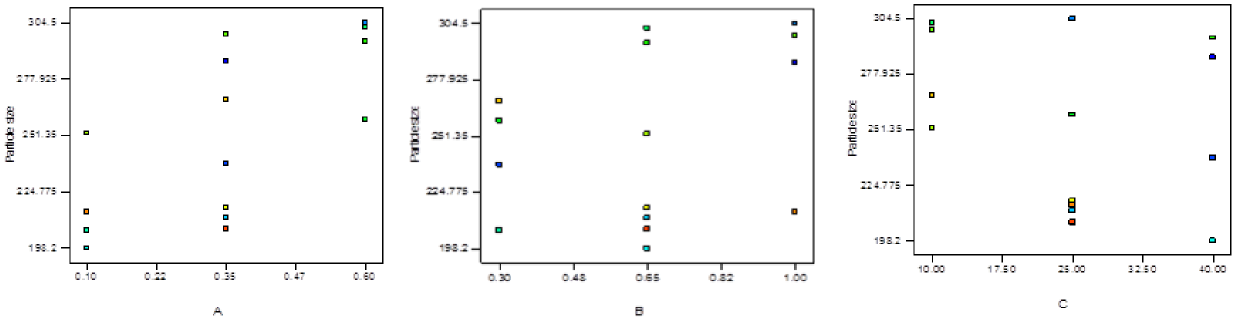

Figure S1. Particle size distribution at different levels of the independent variables (A) TPP (mg/ml) (B) CS (mg/ml) and (C) ultrasonication energy (Watt).

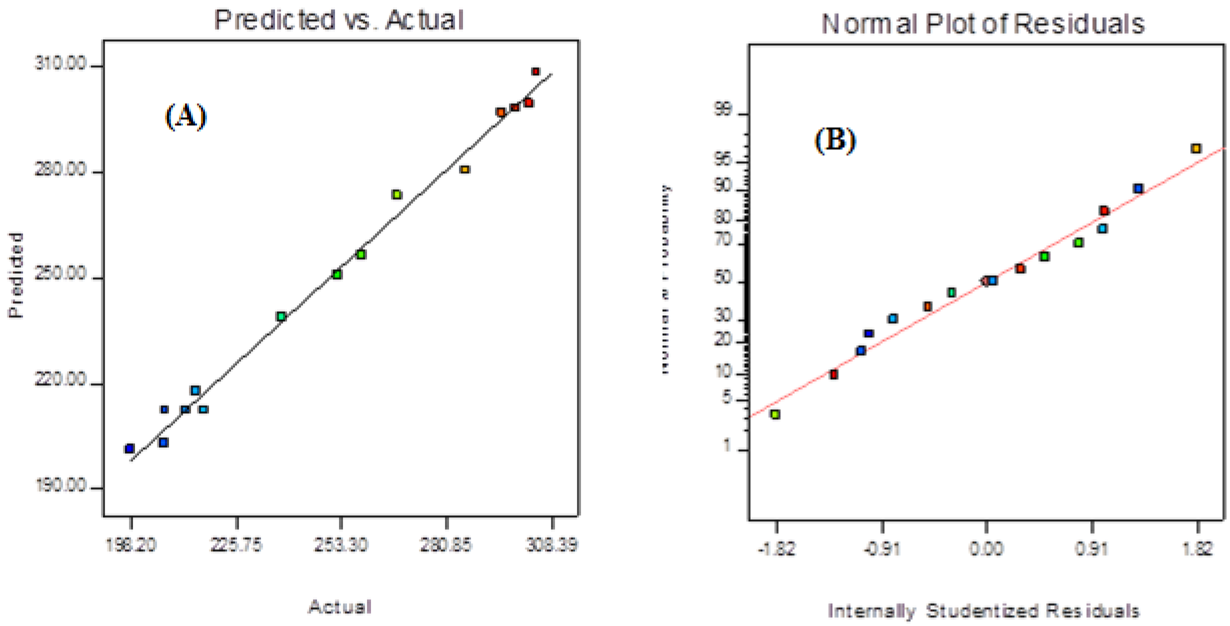

Figure S2. Diagnosis plots: particle size (A) Normal plot of residuals and (B) regression line of response surface plot of actual versus predicted value.

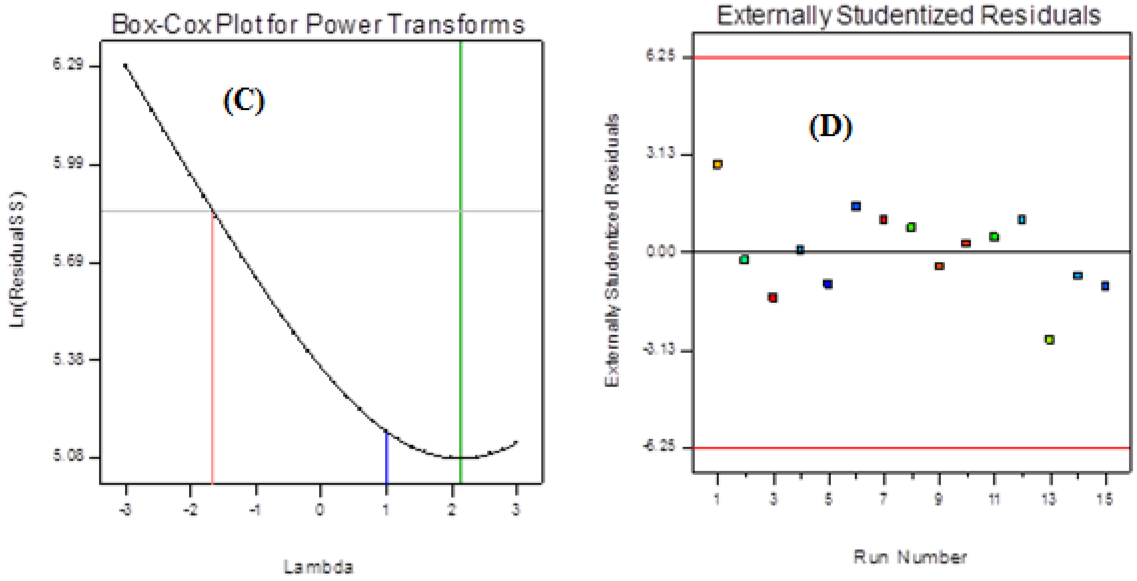

Figure S2. Diagnosis plots; particle size (C) Box Cox plot for power transforms and (D) externally studentized residuals vs. run number.

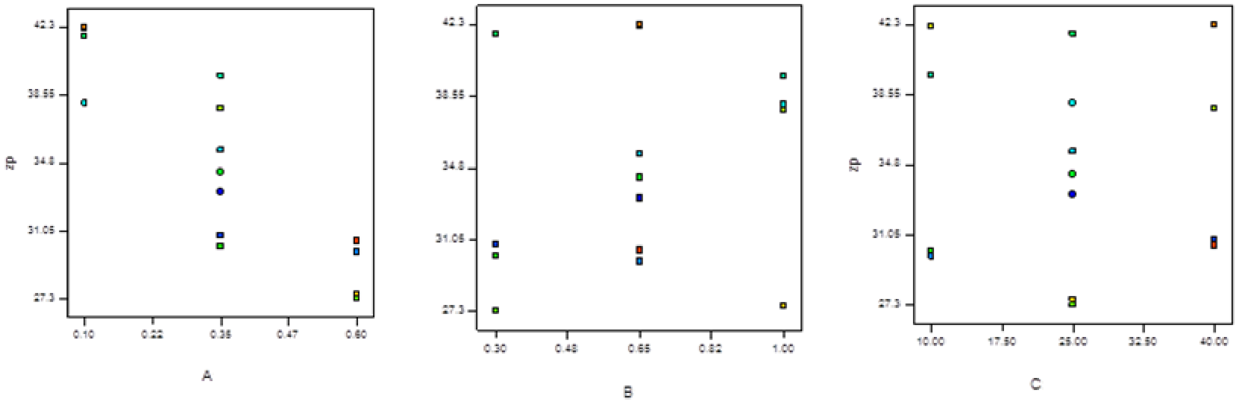

Figure S3. Zeta potential distribution at different levels of the independent variables (A) TPP (mg/ml), (B) CS (mg/ml) and (C) ultrasonication energy (Watt).

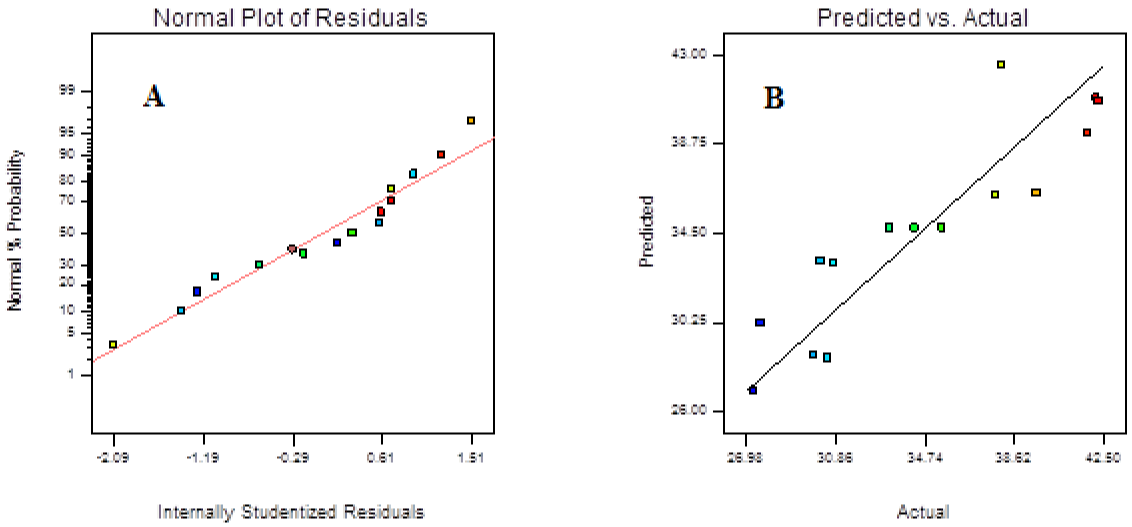

Figure S4. Diagnosis plots; zeta potential (A) normal plot of residuals and (B) regression line of response surface plot of actual versus predicted value.

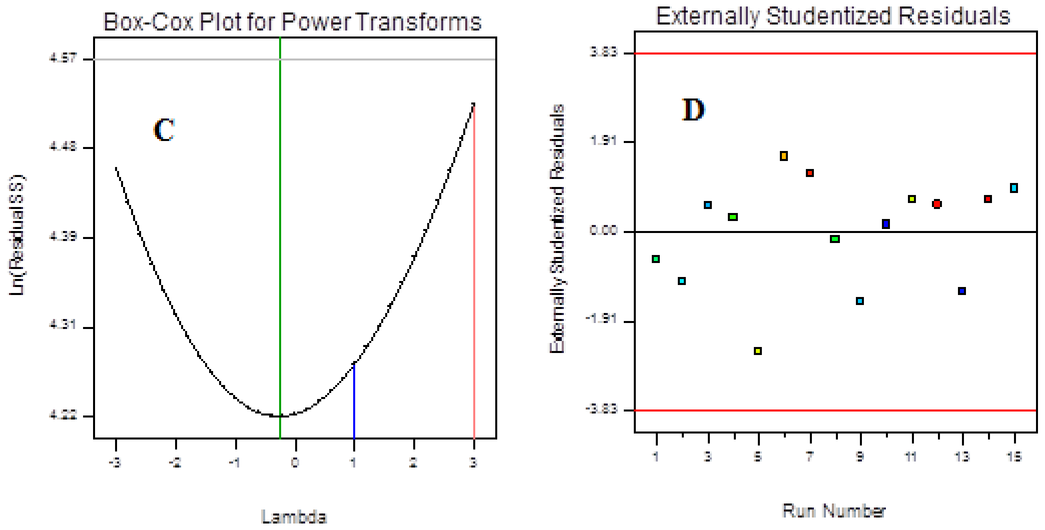

Figure S4. Diagnosis plots; zeta potential (C) Box Cox plot for power transforms and (D) externally studentized residuals vs. run number.

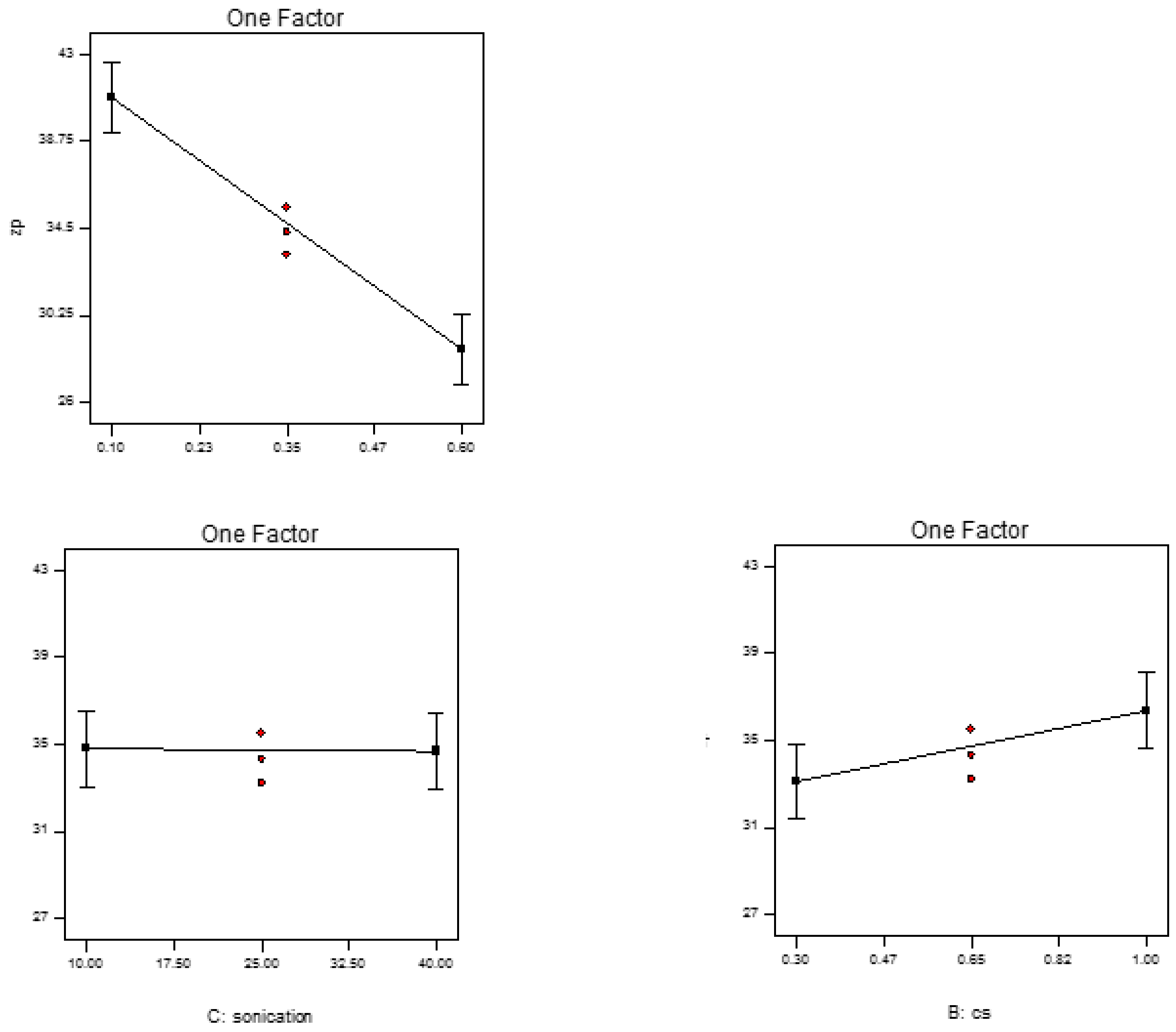

Figure S5. Effect of variables on zeta potential (A: top left) TPP concentraion (B: bottom right) CS concentration and (C: bottom left) ultrasonication energy.

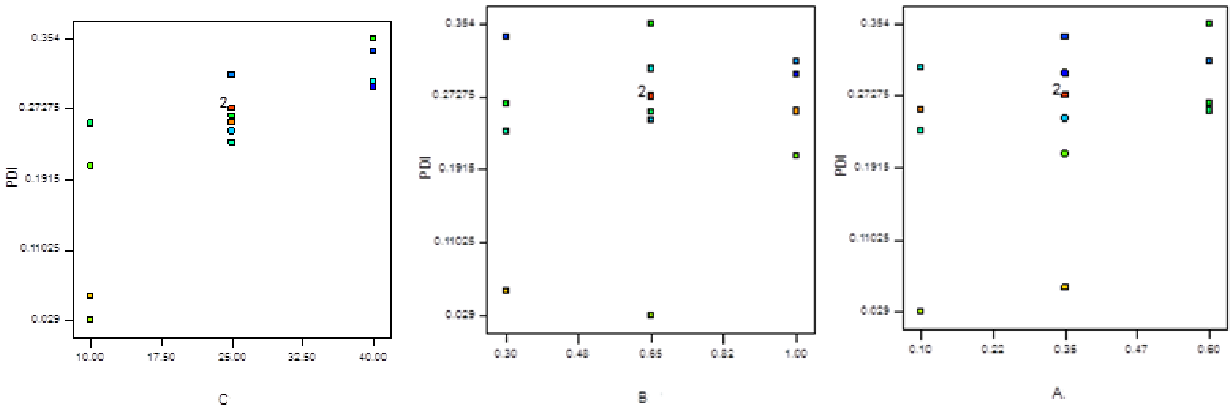

Figure S6. PDI distribution at different levels of the independent variables (A) TPP (mg/ml), (B) CS (mg/ml) and (C) ultrasonication energy (Watt).

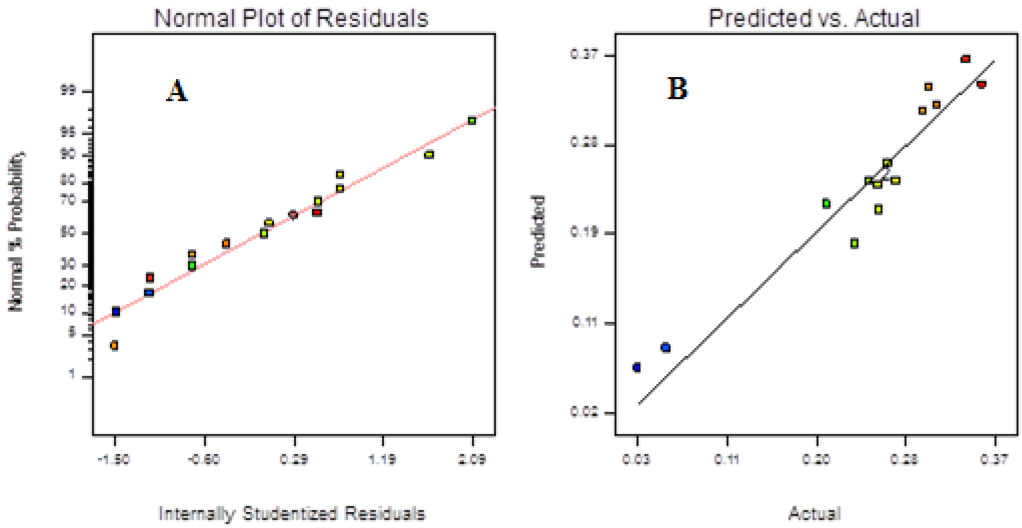

Figure S7. Diagnosis plots: PDI (A) normal plot of residuals and (B) regression line of response surface plot of actual versus predicted value.

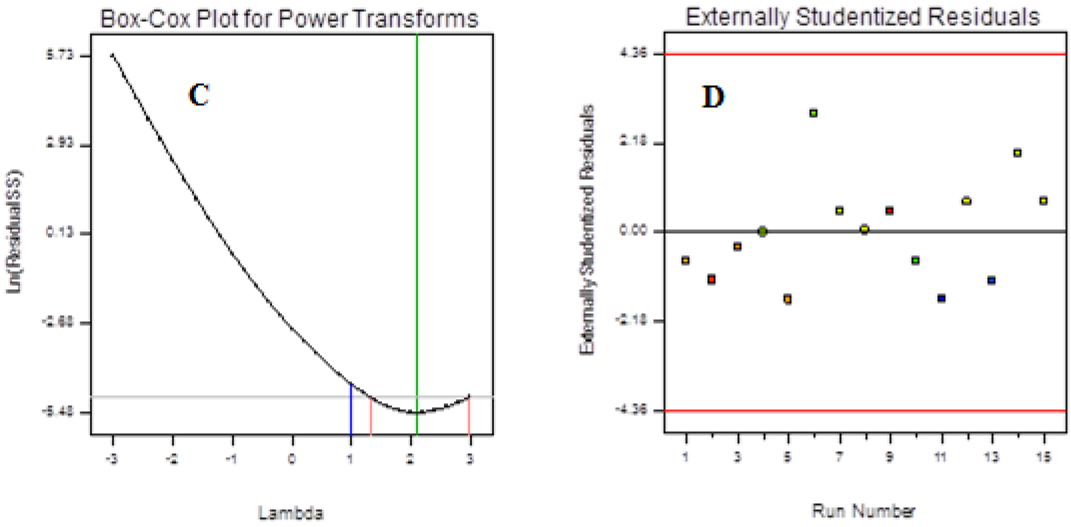

Figure S7. Diagnosis plots: PDI (C) Box Cox plot for power transforms and (D) **e**xternally studentized residuals vs. run number.

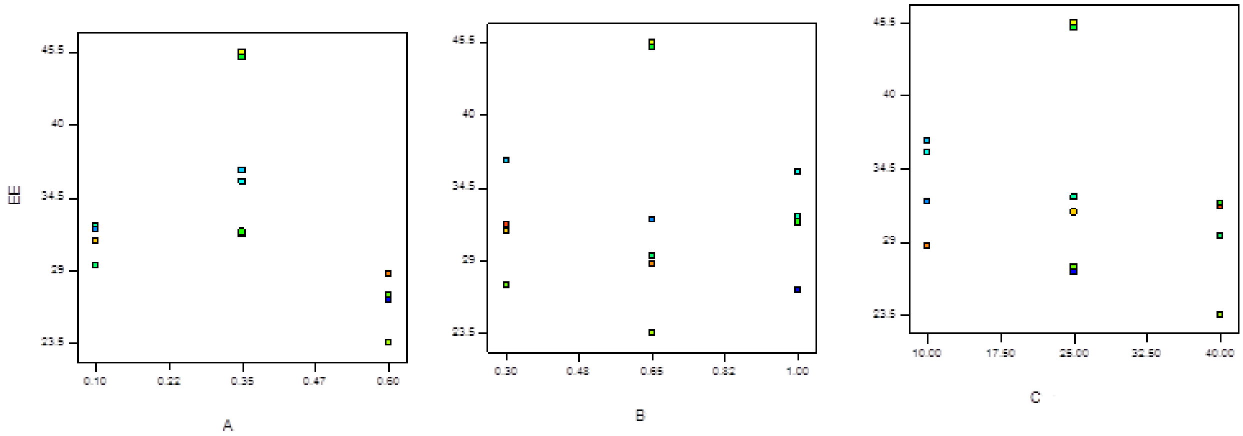

Figure S8. EE distribution at different levels of the independent variables (A) TPP (mg/ml), (B) CS (mg/ml) and (C) ultrasonication energy (Watt).

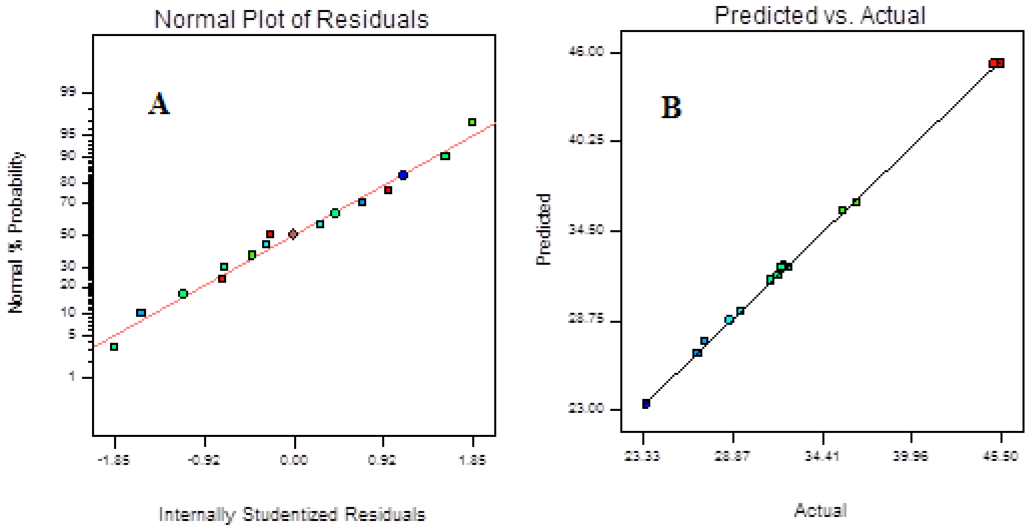

Figure S9. Diagnosis plots: EE (A) normal plot of residuals and (B) regression line of response surface plot of actual versus predicted value.

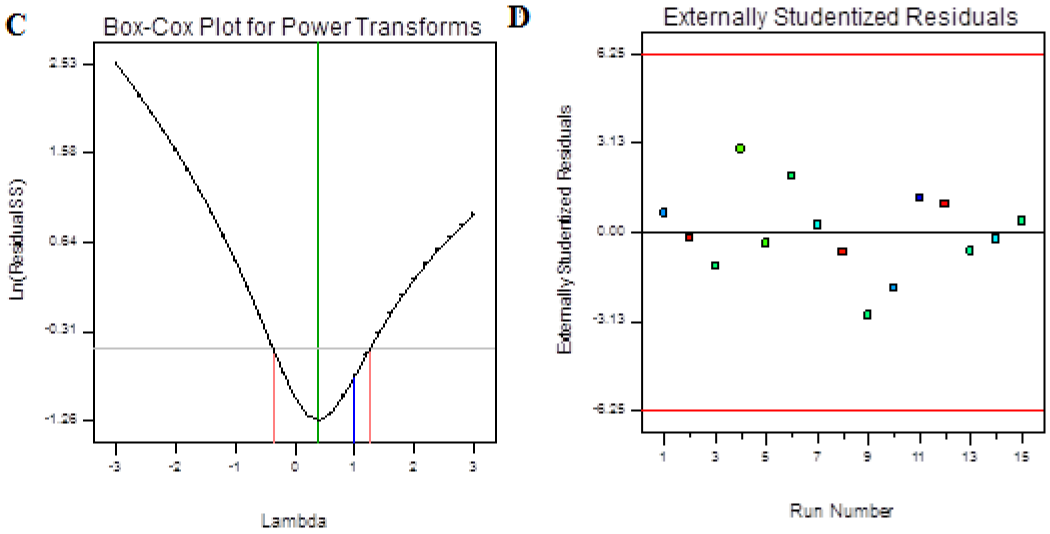

Figure S9. Diagnosis plots: EE (C) Box Cox plot for power transforms and (D) externally studentized residuals vs. run number.

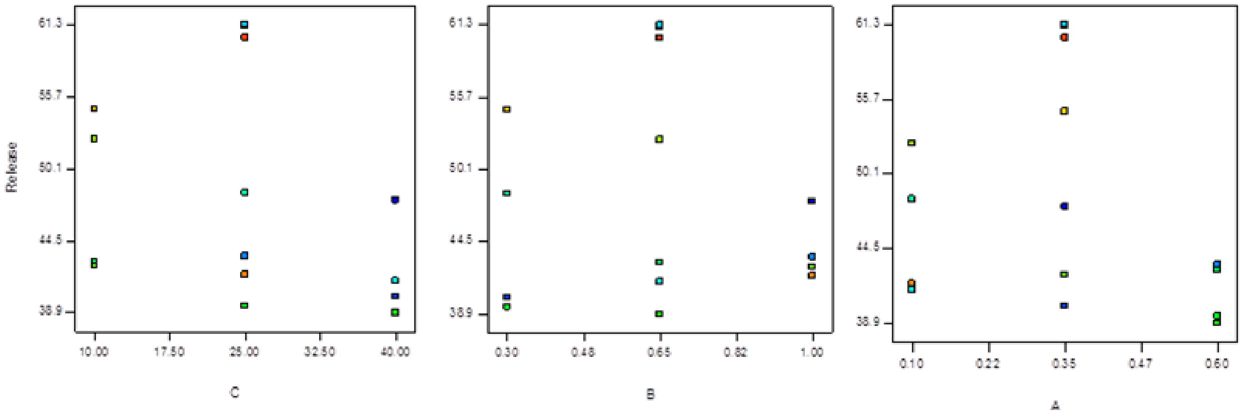

Figure S10. Release distribution at different levels of the independent variables (A) TPP (mg/ml) (B) CS (mg/ml) and (C) ultrasonication energy (Watt).

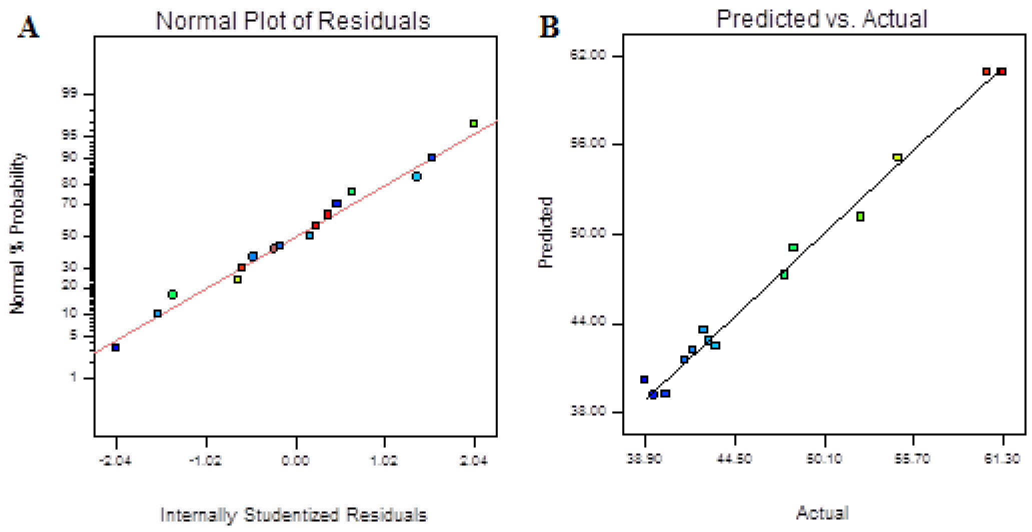

Figure S11. Diagnosis plots: release of CHCl (A) normal plot of residuals and (B) regression line of response surface plot of actual versus predicted value.

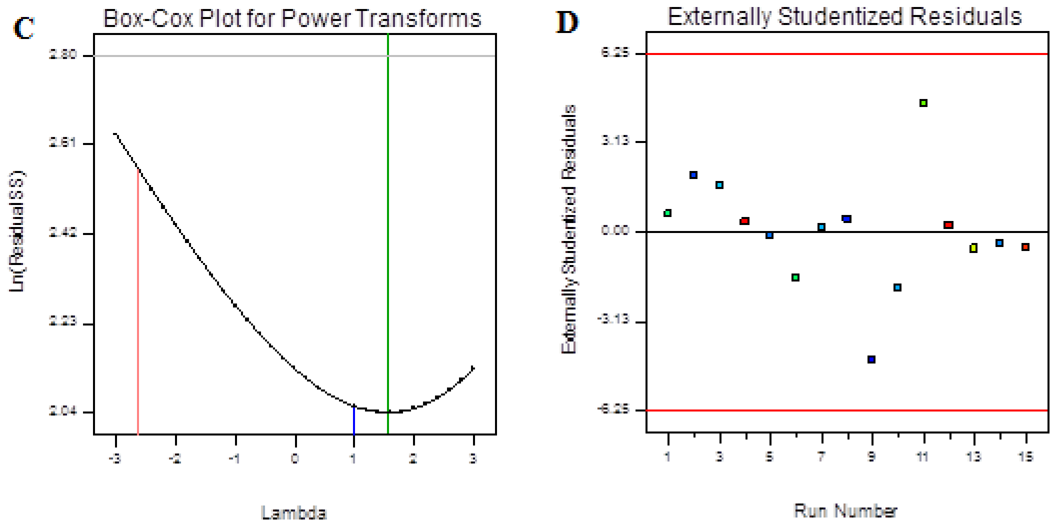

Figure S11. Diagnosis plots: release of CHCl (C) Box Cox plot for power transforms and (D) **e**xternally studentized residuals vs run number.

Table S1. Model summary statistics; response: particle size.

Source	Std. Dev.	R^2	Adjusted R^2	Predicted R^2	PRESS	
Linear	27.11	0.6337	0.5338	0.4168	12870.53	
2FI	29.80	0.6780	0.4365	0.1312	19171.96	
Quadratic	5.92	0.9921	0.9778	0.9050	2096.38	Suggested

"Model Summary Statistics": Focus on the model maximizing the "Adjusted R-Squared and the "Predicted R-Squared".

Table S2. Summary of the results of the regression analysis after fitting to the quadratic model.

STDEV	5.92	R^2	0.9921
Mean	250.78	Adj R^2	0.9778
C.V. %	2.36	Pred R^2	0.9050
PRESS	2096.38	Adeq Precision	22.190

Table S3. Model summary statistics; response: zeta potential.

Source	STDEV	R^2	Adjusted R^2	Predicted R^2	PRESS	
Linear	2.55	0.8187	0.7692	0.6196	149.85	Suggested
2FI	2.87	0.8326	0.7071	0.1421	337.96	
Quadratic	3.23	0.8675	0.6289	-1.0279	798.87	

"Model Summary Statistics": Focus on the model maximizing the "Adjusted R-Squared and the "Predicted R-Squared".

Table S4. Summary of the results of the regression analysis after fitting to the linear model.

STDEV	2.55	R^2	0.8187
Mean	34.74	Adj R^2	0.7692
C.V. %	7.34	Pred R^2	0.6196
PRESS	149.85	Adeq Precision	11.798

Table S5. Model summary statistics; response: PDI.

Source	STDEV	R^2	Adjusted R^2	Predicted R^2	PRESS	
Linear	0.050	0.7675	0.7041	0.5136	0.057	
2FI	0.036	0.9137	0.8489	0.5885	0.048	Suggested
Quadratic	0.032	0.9557	0.8760	0.3438	0.077	

Model Summary Statistics”: Focus on the model maximizing the “Adjusted R-Squared and the “Predicted R-Squared”.

Table S6. Summary of the results of the regression analysis after fitting to the two factor interaction model.

STDEV	0.036	R^2	0.9137
Mean	0.25	Adj R^2	0.8489
C.V. %	14.43	Pred R^2	0.5885
PRESS	0.048	Adeq Precision	12.403

Table S7. Model summary statistics; response encapsulation efficiency.

Source	STDEV	R^2	Adjusted R^2	Predicted R^2	PRESS	
Linear	7.33	0.1186	-0.1218	-0.2742	854.01	
2FI	8.57	0.1223	-0.5359	-1.0950	1404.09	
Quadratic	0.30	0.9993	0.9981	0.9911	5.95	Suggested

"Model Summary Statistics": Focus on the model maximizing the "Adjusted R-Squared" and the "Predicted R-Squared

Table S8. Summary of the results of the regression analysis after fitting to the quadratic model.

STDEV	0.30	R^2	0.9993
Mean	33.52	Adj R^2	0.9981
C.V. %	0.90	Pred R^2	0.9911
PRESS	5.95	Adeq Precision	89.304

Table S9. Model summary statistics; response: release.

Source	STDEV	R^2	Adjusted R^2	Predicted R^2	PRESS	
Linear	8.60	0.1361	-0.0995	-0.3007	1225.28	
2FI	9.21	0.2792	-0.2614	-0.5285	1439.84	
Quadratic	1.25	0.9918	0.9769	0.8768	116.05	Suggested

Table S10. Summary of the results of the regression analysis after fitting to the quadratic model.

STDEV	1.25	R^2	0.9918
Mean	47.75	Adj R^2	0.9769
C.V. %	2.61	Pred R^2	0.8768
PRESS	116.05	Adeq Precision	21.424

Table S11. Optimized formula variables.

A	B	C	Particle size	PDI	ZP	EE	Release	Desirability
0.25	0.65	25	204.627	0.223	33.37	45.1639	60.965	0.970

Table S12. Predicted Vs. actual responses obtained for the optimized formulation.

Predicted	204.627	0.223	33.37	45.1639	60.965
Actual	209.25±13.45	0.235± 0.09	32.03± 0.012	41.75± 3.246	59.8±9.41
%Error	2.25	5.381	-4.015	-7.55	-1.91
